# Prominent mediatory role of gut microbiome in the effect of lifestyle on host metabolic phenotypes

**DOI:** 10.1080/19490976.2025.2599565

**Published:** 2025-12-17

**Authors:** Solia Adriouch, Eugeni Belda, Timothy D. Swartz, Sofia Forslund, Edi Prifti, Judith Aron-Wisnewsky, Rima Chakaroun, Trine Nielsen, Christine Poitou, Pierre Bel-Lassen, Christine Rouault, Tiphaine Le Roy, Petros Andrikopoulos, Kanta Chechi, Francesc Puig-Castellví, Inés Castro Dionicio, Philippe Froguel, Bridget Holmes, Rohia Alili, Fabrizio Andreelli, Hedi Soula, Joe-Elie Salem, Gwen Falony, Sara Vieira-Silva, Jeroen Raes, Peer Bork, Michael Stumvoll, Oluf Pedersen, S. Dusko Ehrlich, Marc-Emmanuel Dumas, Jean-Michel Oppert, Maria Carlota Dao, Jean-Daniel Zucker, Karine Clément

**Affiliations:** a Sorbonne Université, Inserm, Nutrition and Obesities: Systemic Approaches, NutriOmics, Research Unit, Paris, France; b Sorbonne Université, IRD, Unité de Modélisation Mathématique et Informatique des Systèmes Complexes, UMMISCO, Bondy, France; c Integrative Phenomics, Paris, France; d Charité-Universitätsmedizin Berlin, Freie Universität Berlin and Humboldt-Universität zu Berlin, Berlin, Germany; e Max Delbrück Center for Molecular Medicine in the Helmholtz Association (MDC), Berlin, Germany; f Experimental and Clinical Research Center, Max Delbrück Center for Molecular Medicine and Charité-Universitätsmedizin Berlin, Berlin, Germany; g DZHK (German Centre for Cardiovascular Research), partner site Berlin, Berlin, Germany; h Structural and Computational Biology Unit, EMBL, Heidelberg, Germany; i Assistance Publique – Hopitaux de Paris, Department of Nutrition, Pitié-Salpêtrière Hospital, Sorbonne University, Paris, France; j Wallenberg Laboratory, Department of Molecular and Clinical Medicine and Sahlgrenska Center for Cardiovascular and Metabolic Research, University of Gothenburg, Gothenburg, Sweden; k Medical Department III - Endocrinology, Nephrology, Rheumatology, University of Leipzig Medical Center, Leipzig, Germany; l Novo Nordisk Foundation Center for Basic Metabolic Research, Faculty of Health and Medical Science, University of Copenhagen, Copenhagen; m Steno Diabetes Center Copenhagen, Herlev, Denmark; n Department of Clinical Medicine, University of Copenhagen, Copenhagen, Denmark; o Division of Systems Medicine, Department of Metabolism, Digestion and Reproduction, Imperial College London, London, UK; p EGENODIA Inserm U1283, CNRS UMR8199, Institut Pasteur de Lille, Lille University Hospital, European Genomic Institute for Diabetes (EGID), Université de Lille, Lille, France; q FAO, Food and Nutrition Division, Rome, Italy; r Assistance Publique – Hopitaux de Paris, Department of Diabetology, Pitié-Salpêtrière Hospital, Paris, France; s Assistance Publique – Hôpitaux de Paris (AP-HP), Clinical Investigation Center (CIC-1901), Department of Pharmacology, Pitié-Salpêtrière Hospital, Sorbonne Université, Institut National de la Santé et de la Recherche Médicale (INSERM), Paris, France; t Host Microbe Interactomics Group, Wageningen University & Research, Wageningen, The Netherlands; u Institute of Medical Microbiology and Hygiene and Research Center for Immunotherapy (FZI), University Medical Center of the Johannes Gutenberg-University Mainz, Mainz, Germany; v Institute of Molecular Biology (IMB), Mainz, Germany; w VIB Center for Microbiology, VIB, Leuven, Belgium; x EMBL, Heidelberg, Germany; y Helmholtz-Institute of Metabolism, Obesity and Vascular Research (HI-MAG) at Leipzig University, Helmholtz Munich, Germany; z Center for Clinical Metabolic Research, Herlev-Gentofte University Hospital, Copenhagen, Denmark; aa Department of Medicine, University of Copenhagen and Herlev-Gentofte University Hospital, Copenhagen, Denmark; ab Department of Clinical and Movement Neurosciences, UCL Queen Square Institute of Neurology, London, UK; ac Université Paris-Saclay, INRAE, MGP, Jouy-en-Josas, France; ad Victor Phillip Dahdaleh Institute of Genomic Medicine, McGill University, Montréal, QC, Canada; ae Department of Agriculture, Nutrition, and Food Systems, University of New Hampshire, Durham, NH, USA

**Keywords:** Gut microbiome, nutrition, lifestyle, mediation

## Abstract

Lifestyle factors influence both gut microbiome composition and host metabolism, yet their combined and mediating effects on host phenotypes remain poorly characterized in cardiometabolic populations. In 1,643 participants from the MetaCardis study, we developed a composite lifestyle score (QASD: dietary quality, physical activity, smoking, and diet diversity) that outperformed individual lifestyle variables in explaining microbial gene richness and exhibited a significant impact on the gut microbiome composition. While bidirectional pathways linking the QASD score, host phenotypes, and microbiome composition were assessed, causal inference-based mediation analyses indicated stronger effects when the microbiome was modeled as the mediator variable, particularly in relation to the insulin resistance-associated profile. Microbiome gene richness emerged as a key mediator explaining 27.8% of QASD score’s effect on the insulin resistance marker (HOMA-IR), while no significant mediation was observed on BMI. Extended mediation analyses on microbial species and serum metabolomics deconfounded for drug use and clinical profiles identified 47 mediations where microbial taxa mediated more than 20% of the effect of the QASD score on serum metabolites associated with insulin resistance. Notably, several *Faecalibacterium* lineages enriched in individuals with high QASD score played a significant mediatory role in increasing the serum biomarkers of microbiome diversity (as cinnamoylglycine or 3-phenylpropionate). Conversely, elevated levels of secondary bile acids in individuals with low QASD scores were strongly mediated by high levels of *Clostridium bolteae*. These findings highlight distinct and clinically relevant microbiome pathways linking lifestyle behaviors to cardiometabolic risks.

One sentence summary:

The gut microbiome mediates the impact of diet quality and diversity, physical activity and smoking status – combined in a composite lifestyle score – on cardiometabolic phenotypes.

## Introduction

Various medical conditions underpinned by chronic systemic inflammation and insulin resistance, from type 2 diabetes and obesity^1-3^ to cardiovascular diseases,^4^ have been consistently associated with alterations in the gut microbiota. In these diseases, an imbalance in microbial populations is characterized by reduced gut microbial gene richness (GMGR) and diversity, along with significant changes in microbial composition and function.^5^

Reports regularly describe the importance of a wide array of lifestyle factors in the onset and progression of cardiometabolic diseases, also suggesting a potential mediating role of the gut microbiome in these relationships.^6^^,^^7^ However, while many studies have reported associations in human populations, only a few have sought to characterize microbiome features that may mechanistically mediate the effects of lifestyle patterns on disease pathophysiology and phenotypes. Among modifiable lifestyle factors, dietary patterns, physical activity, sedentary behavior, and smoking are well-established contributors to cardiometabolic health, that may act either directly or through the modification of gut microbial composition and function. ^8^^,^^9^

Dietary habits are commonly evaluated based on the intake of specific food items, considering their protective or harmful effects.^10^ To capture dietary patterns in relation to health outcomes, several scoring systems have been developed.^10^ Among these, the alternative healthy eating index (AHEI) and the dietary approaches to stop hypertension (DASH) score are widely used to assess diet quality and have been linked to reduced cardiovascular risks.^11^ On the other hand, the alternative dietary inflammatory index (aDII),^12^ capture the inflammatory potential of the diet. Few studies have systematically examined the relationship between these score-based dietary patterns and the gut microbiome composition and potential interactions with cardiometabolic phenotypes.^13-15^

Another component of food consumption is diet diversity (commonly defined by the number of distinct food groups) that is widely recommended to ensure nutrient adequacy.^16^ However, dietary diversity is not necessarily indicative of overall diet quality.^17^ Commonly used dietary diversity scores rarely account for the distribution of food items within each food groups, referred to here as dietary variety.^17^ As such, ecological diversity indices can address these aspects, providing a framework for evaluating the interplay between dietary patterns, the gut microbiome^18^ and clinical phenotypes. Previous studies have focused on healthy or elderly populations. For instance, one study reported a positive association between dietary diversity and longitudinal microbial stability in 34 healthy individuals but found no relationship with microbiome richness.^19^ The PREDICT 1 study demonstrated that fecal microbiome diversity was associated with habitual dietary intake and diversity in the general population.^7^ Another study in 445 elderly subjects employed a diversity score similar to the Simpson index, linking a more diverse diet to a more even distribution of bacterial groups taxa.^20^ However, few studies have examined how dietary diversity, independent of dietary quality, may be related to the gut microbiome composition and metabolic health across varying degrees of cardiometabolic disease severity.^7^^,^^19^^,^^20^

Other lifestyle factors are important for cardiometabolic health. For instance, the Global Burden of Disease Study report (2017) highlights the substantial health risks associated not only with inadequate fruit and vegetable consumption,^21^ but also with alcohol intake, smoking, and insufficient physical activity.^21^ Alcohol consumption has been shown to differentially affect the gut microbiome composition depending on the presence of underlying disease.^7^^,^^22^^,^^23^ Similarly, physical activity^24^ and smoking status^25^ have been shown to significantly modify the gut microbiome composition. This suggests that integrative lifestyle indices accounting for these factors should be of interest when investigating the microbiome composition and its links with host phenotypes. However, no study to date has examined the combined effects of physical activity and smoking status alongside dietary indices,^26^ in relation to the gut microbiome and metabolic health.^27^^,^^28^ In particular, it remains to be identified whether a composite lifestyle score combining these lifestyle factors would better capture these associations with the microbiome than individual factors and whether specific microbiome features may partly mediate the effect of lifestyle factors on metabolic phenotypes.

We addressed these aspects in 1,643 participants from the European MetaCardis population encompassing a broad spectrum of cardiometabolic phenotypes and developed a composite lifestyle score, named the QASD, that includes diet quality, physical activity, smoking status, and dietary diversity, to examine its associations with metabolic health phenotypes, notably insulin resistance markers, the serum metabolome, and the fecal microbiome composition in comparison with individual lifestyle features. In addition, we performed bidirectional mediation analyses, providing novel insights into the complex, interrelated pathways linking lifestyle behaviors, the gut microbiome, and cardiometabolic risks.

## Materials and methods

### Study cohort and sample acquisition

This study is based on the European MetaCardis cohort, which was originally designed to investigate the relationships between different stages of disease and gut microbiome variation. The cross-sectional MetaCardis cohort included 2,214 participants, covering a wide range of metabolic and cardiac phenotypes. Participants were recruited between 2013 and 2015 from Denmark, Germany, and France. Details regarding the study cohort, including protocol information, exclusion criteria, group definitions, biochemical analyses, and the collection of anthropometric and clinical data, have been extensively described in previous publications.^2^^,^^29^^,^^30^

For the present study, we focused on a subset of 1,643 individuals, including healthy, normal-weight participants (BMI <25 kg/m²), as well as individuals with various metabolic conditions, such as type 2 diabetes (T2D), metabolic syndrome (MS), obesity and cardiovascular disease. This subset is summarized in the flow chart in Supplementary Figure 1 and detailed in Supplementary Table S1. The study protocol was approved by the Ethics Committees of the Medical Faculty at the University of Leipzig, Germany (application number: 047-13-28012013), the Capital Region of Denmark (H-3-2013-145), and the ‘Comité de Protection des Personnes’ (CPP) Ile-de-France III, France (no. IDRCB2013-A00189-36). The study was registered at https://clinicaltrials.gov/ (NCT02059538). The cohort design adhered to ethical regulations, including the Declaration of Helsinki and European privacy laws. All participants provided written informed consent.

As a replication cohort for individuals with overweight and obesity, we used data from the GutInside study (see Supplementary Table S2) described in.^31^ Participants were recruited in France from September 2018 to January 2020 across various regions and included 433 individuals with overweight or obesity (BMI ≥ 25 kg/m²). Although the GutInside participants were involved in a dietary intervention program, only baseline data were analyzed for this study. Similar questionnaires and food records were used to evaluate lifestyle factors.

#### Clinical and anthropometric variables and definition of study groups

Study groups were defined according to international disease criteria. Overweight and obesity were classified using the WHO criteria, with overweight defined as BMI ≥25 kg/m² and obesity defined as BMI ≥30 kg/m². MS was determined based on the International Diabetes Federation’s 2005 Consensus Worldwide Definition (http://www.idf.org/metabolic-syndrome), which requires a waist circumference >94 cm in men and >80 cm in women, along with any two of the following four factors: (i) triglycerides ≥1.7 mmol/L or treatment for lipid abnormalities (e.g., statins, fibrates, or ezetimibe); (ii) high-density lipoprotein (HDL) cholesterol <1.03 mmol/L in men and <1.29 mmol/L in women, or treatment for lipid abnormalities; (iii) blood pressure (BP) ≥130/85 mmHg, or treatment with antihypertensive medication; and (iv) fasting plasma glucose ≥5.6 mmol/L or a diagnosis of T2D. T2D was defined using the American Diabetes Association (ADA) criteria: fasting glucose >6.9 mmol/L, 2-h values during an oral glucose tolerance test >11 mmol/L, HbA1c (glycated hemoglobin) ≥6.5%, or the use of anti-diabetic medication (American Diabetes Association, 2018). Hypertension status was defined according to the American College of Cardiology and American Heart Association guidelines.^32^

For obesity, participants were categorized into two groups: Group 2A: individuals with Grade II obesity (BMI ≥35 kg/m²) without T2D or previous cardiovascular conditions. Group 2B: Predominantly individuals with Grade III obesity (BMI ≥40 kg/m²) eligible for bariatric surgery. T2D was not an exclusion criterion, and participants generally exhibited more severe metabolic impairments compared to Group 2A. For descriptive purposes, Groups 2A and 2B were combined in this study. Participants with heart failure were defined according to guidelines from the American College of Cardiology, American Heart Association, and Heart Failure Society of America.^33^ The group with coronary heart disease included patients with first events of acute coronary syndromes, chronic coronary artery disease with or without heart failure (defined as left ventricular ejection fraction ≥45% or <45%, respectively). Participants with heart failure due to non-coronary causes were excluded.

Anthropometric measurements, including weight, height and waist circumference, were obtained during clinical visits using standardized procedures and calibrated equipment. Body fat mass and fat-free mass were assessed by bioelectrical impedance analysis. Systolic and diastolic blood pressure were measured using a mercury sphygmomanometer, with three readings taken on each arm; the average of the last two measurements from the right arm was used for analysis. A detailed record of prescribed medications and the patient’s medical history was collected, with drug treatments categorized according to their molecular class, as described previously.^29^

## Dietary intake and lifestyle data assessment

### General questionnaire and database

In the MetaCardis EU project, a clearly defined objective was to study the interplay between lifestyle, diet, the fecal metagenome and host metabolic conditions. Dietary data for the MetaCardis population were collected using a web-based, validated food-frequency questionnaire (FFQ) tailored to the cultural habits of each recruitment country. The MetaCardis FFQ was adapted from the validated European Prospective Investigation of Cancer (EPIC)-Norfolk FFQ and incorporated elements from several other European FFQs. The French version of the FFQ version contains 159 food and beverage items, the German FFQ contains 143 items, and the Danish FFQ contains 153 items. The FFQ asked participants to report the frequency of consumption of each food item over the last 12 months, using a 9-level scale, ranging from “never or less than once a month” to “6 times or more per day”. For each food item, a generic portion size was specified. Thus, initial responses to the FFQ were frequency choices indicating consumption units per month, week, or day for each food items. These frequencies were converted to daily intake values and then translated into food group intakes (in g/day) and nutrient intakes (in g/day, mg/day, or mcg/day) using data on portion sizes (i.e., grams per portion) and nutrient contents (i.e., nutrients per portion) sourced from nationally relevant food composition databases. A validation study comparing the FFQ against repeated 24-h dietary records among 324 French MetaCardis participants showed good validity for micronutrient intake.^34^

Several compounds and nutrients have been added to the original composition table, allowing for more precise analysis of specific compounds, such as subclasses and totals of polyphenols (using the updated Phenol-Explorer table^35^) and nutrients/metabolites from updated databases, including the USDA (https://fdc.nal.usda.gov/), FooDB (https://foodb.ca/), and the Danish National Food Database (DTU Food Database: https://frida.fooddata.dk/) for biotin intake^3^. Each nutrient or metabolite of interest was carefully integrated into a consortium internal composition table through dietary matching and detailed recipe decomposition, which was performed by dietitians and experts. The basal metabolic rate (BMR) for each participant was estimated using the Harris and Benedict equation. Participants who significantly under- or overreported energy intake (defined as <0.5 BMR or >3.5 BMR) were excluded from the dietary analysis (less than 10% of the subjects presented nutritional data).

Physical activity and sedentary behavior data were collected using the Recent Physical Activity Questionnaire (RPAQ).^33^ The RPAQ assesses habitual physical activity over the past month across four domains: home, work, travel, and leisure time. Physical activity assessment using the RPAQ has been validated against energy expenditure measurements using the doubly-labeled water method.^36^ The physical activity data were computed as hours per week and as the metabolic equivalent of task (MET)-hour per week, using a published compendium of physical activities and their corresponding MET values. One MET represents the energy expended at rest, equivalent to 3.5 ml/min/kg of oxygen consumption.^37^ Sedentary behavior was assessed through leisure screen time, which represents the sum of time spent watching TV and using the computer during leisure time, expressed in hours/week.

Smoking status data were obtained through multiple questions regarding smoking habits, including the subject’s smoking status (non-smoker, former smoker, passive smoker, current smoker), the number of cigarettes smoked, and the date of cessation or resumption of smoking. For the QASD score calculations, smoking status was recoded into two categories (non-smoker/smoker), as shown in Supplementary Table S1. The initial smoking status classification was cross-checked with quantitative data on cigarette consumption, start/stop dates, and passive smoking exposure to confirm the final categorization (non-smoker/smoker).

### Food groups and nutrient content

Food items from country-specific FFQ were combined into a list of 160 food items. Food group consumption was calculated by organizing the 160 food items into groups of nutritional interest. The original 22 groups in the MetaCardis study were further divided into 42 subgroups (as shown in Supplementary Table S3, diversity 1 column). The dietary diversity scores were based on these 42 subgroups, following specific criteria. For sensitivity analysis, dietary diversity scores were also constructed by excluding “liquid” foods, such as hot drinks, fruit juices, sugary drinks, alcoholic beverages, and milk, as their quantities could disproportionately affect the diversity scores based on consumption uniformity (Simpson index).

#### Dietary “inflammatory potential” of the diet

The “inflammatory potential” of the diet was assessed using the alternative dietary inflammatory index (aDII), an adapted version of the original dietary inflammatory index (DII) developed by Cavicchia et al.^12^ and based on the methodology outlined by van Woudenbergh et al.^38^ The aDII includes 34 food items, standardized after adjusting for energy intake using the residual method developed by Willett et al.^39^ to minimize variations in dietary intake due to differences in physical activity, body size, and metabolic efficiency. Each food item's value was then multiplied by a literature-based inflammatory weight that reflected the pro/anti-inflammatory potential of the food item,^12^ and the resulting values were summed to calculate the aDII score. Higher aDII scores indicate a more proinflammatory diet.

### Dietary quality scores

The alternative healthy eating index (AHEI) and dietary approaches to stop hypertension (DASH) scores were computed to evaluate overall dietary quality, and two *a priori* scores, taking into account the existing correlations between the different components of the diet developed in order to overcome the limitations linked to approaches focusing only on food or nutrient groups^40^ and based on foods and nutrients predictive of chronic disease risk.^41^^,^^42^ As a measure of healthy US-style eating, the AHEI-2010 assigns 0–10 points to each of 11 dietary components based on the portion size.^40^^,^^41^ The DASH score contains 8 components, each of which receives 1–5 points according to its consumption quintile.^43^

**Dietary diversity and variety scores:** Dietary diversity and variety scores were constructed based on different food group categorizations (42 groups aggregated, with or without “liquid” foods, as shown in Supplementary Table S3) and two different estimation methods: count-based and evenness-based.

**Count-based scores:** We employed count-based measures of diversity, which record the number of distinct food items and groups consumed, without considering the quantities of each consumed food. Simple count-based scores were calculated by assigning 0 points for each food group (for diversity scores) or individual food item (for variety scores) if the participant did not consume the food (consumption = 0 grams) and 1 point if the food was consumed (consumption > 0 grams). Thus, the total diet diversity score ranged from 0 to 42, based on the initial food groups derived from the FFQ (Supplementary Table S3 for the initial 42 groups). While count measures simply tally different food groups and subgroups, they do not account for the distribution of food quantities. Previous research has been limited by these count-based measures and inconsistencies in the number and types of food groups considered.^17^ To address these limitations, we conducted sensitivity analyses, systematically varying the number of food groups and excluding liquid foods from the diet.

**Evenness-based scores:** We also used diversity measures that account for both the number and the distribution of different food items, such as the Berry index, also known as the Simpson index,^44^ which is commonly applied to assess metagenomic or ecological diversity. This index increases when food items are consumed more equally rather than when they are concentrated in fewer groups. Simpson-based scores are calculated by treating food groups (for diversity) or individual foods within a group (for variety) as different species, similar to how ecological indices measure species diversity in an environment. The Simpson index considers both the number of “species” (food groups) present and their relative abundance:

$$\mathrm{Simpson Index}=1-\sum_{i=1}^{R}P_{i}^{2}$$

where *R* represents the richness or number of food groups considered, and *P*_i_^2^ is the relative abundance of each group (the share of food group *i* in the total amount consumed). Subtracting this score from 1 results in an index ranging from 0 to 1, where 0 indicates minimal diversity and 1 represents maximal diversity (equal consumption of all considered items).

To address inconsistencies in the number and types of foods or food groups, we performed sensitivity analyses, varying the groups included in the calculations. Specifically, for total diet diversity, we created scores that excluded liquid foods (see Supplementary Table S3 for additional food groups excluding beverages and milk). We calculated the healthy food diversity (HFD) score developed by Drescher et al.,^45^ which serves as an indicator of healthy food diversity, specifically focusing on the diversity of health-promoting products. We also derived a normalized composite dietary diversity and variety score,^46^^,^^47^ which combines both diversity and variety measures (simple counts), normalized on a scale of 0–100.

These scores capture various aspects of dietary diversity and variety. Some scores reflect the entire diet (referred to as “diversity all diet”), while others focus on specific dietary groups or items (labeled as “variety”). We used diversity and variety scores based on simple counts within consumption groups (labeled as “count”) and scores that account for the evenness of consumption within dietary groups (labeled as “Simpson”).

**Quality activity smoking diversity (QASD) lifestyle score construction:** a new lifestyle score, the QASD, was developed to incorporate diet quality, total physical activity, smoking status, and dietary diversity. Lifestyle components on continuous scales were categorized into three levels to enhance interpretability, harmonize variables measured on different scales (e.g., diet quality-diversity, physical activity), while for smoking status, a value was assigned based on binary classification. This approach ensures robustness across cohorts with heterogeneous distributions, which is consistent with approaches used in other lifestyle indices (e.g., the Healthy Lifestyle Index, Life’s Simple 7, Mediterranean Diet Score^41^^,^^48^^,^^49^). The values of the four individual scores used to build the QASD are given below.

- **Diet quality** (AHEI): 0 points for the low tertile, 1 point for the medium tertile, and 2 points for the high tertile.
- **Dietary diversity** (Simpson index, excluding beverages): 0 points for the low tertile, 1 point for the medium tertile, and 2 points for the high tertile.
- **Total physical activity** (MET-h.week-1): 0 points for the low tertile, 1 point for the medium tertile, and 2 points for the high tertile.
- **Smoking status**: 0 points for smokers and 1 point for non-smokers or ancient smokers.

**Figure 1.**
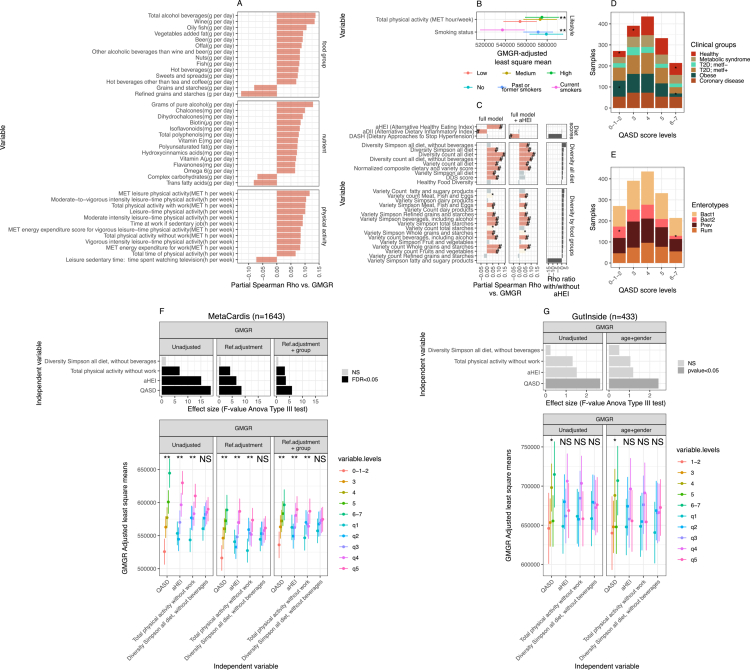
Associations between diet, physical activity factors, and a composite lifestyle score, and gut microbial gene richness (GMGR) are shown. (A) Bar plots showing 40 food groups, nutrients, and physical activity variables significantly associated with the GMGR (FDR < 0.05). Associations are based on partial Spearman's rank correlations adjusted for age, recruitment center, energy intake (kcal), antibiotic treatments (courses in the past 5 y), and the use of metformin, statins, and proton pump inhibitors (PPIs) (MET = metabolic equivalent of task; h per week = hour per week). (B) Associations of lifestyle factors (total physical activity and smoking status) with the GMGR. Physical activity is categorized into tertiles (low/medium/high). The estimated marginal means of the GMGR across these levels are derived from linear regression models adjusted for age, recruitment center, energy intake (kcal), antibiotic treatments, and the use of metformin, statins, and PPIs (FDR < 0.05; ANOVA tests on linear regression models). (C) Associations of dietary scores (AHEI, DASH, and aDII) and 25 dietary diversity and variety scores with the GMGR (*: *p* < 0.05; #: FDR < 0.05; partial Spearman's rank correlation). The fully adjusted model (full model) included adjustments for age, recruitment center, energy intake (kcal), antibiotic treatments, and the use of metformin, statins, and PPIs. The fully adjusted model + AHEI includes additional adjustment by the alternative healthy eating index to evaluate independence from diet quality (AHEI itself excluded in these analyses). Scores are presented for global dietary scores (top), overall diet (middle) and specific food groups (bottom). The variables on the y-axis are ordered by the ratio of partial Spearman correlation coefficients with and without AHEI adjustment (right-most panel). (D,E) Distribution of individuals in the MetaCardis population across levels of the QASD score coloured by clinical group and metagenome-built enterotypes respectively (* = FDR < 0.05, post-hoc analysis for Pearson’s chi-square test for count data). (F) Top bar plots represent the effect sizes (F-values) derived from the ANOVA Type III test on linear regression models where the GMGR (dependent variable) is regressed against the QASD score and its individual components (y-axis, 1 regression model × independent variable) under different adjustment frameworks (Ref. adjustment: age + recruitment center + energy intake (kcal) + antibiotic treatments, and use of metformin + statins + PPIs; group = metacardis clinical groups) in individuals of MetaCardis cohort. The bottom panels represent the estimated marginal means of the GMGR across the QASD score levels and quintiles of the distribution of QASD score individual components derived from the same linear regression models (**: FDR < 0.05, * = *P*-value < 0.05 & FDR > 0.05, NS = non-significant; ANOVA type III test). (G) Similar analyses as panel D were performed in the GutInside cohort (*n* = 433).

**Figure 2.**
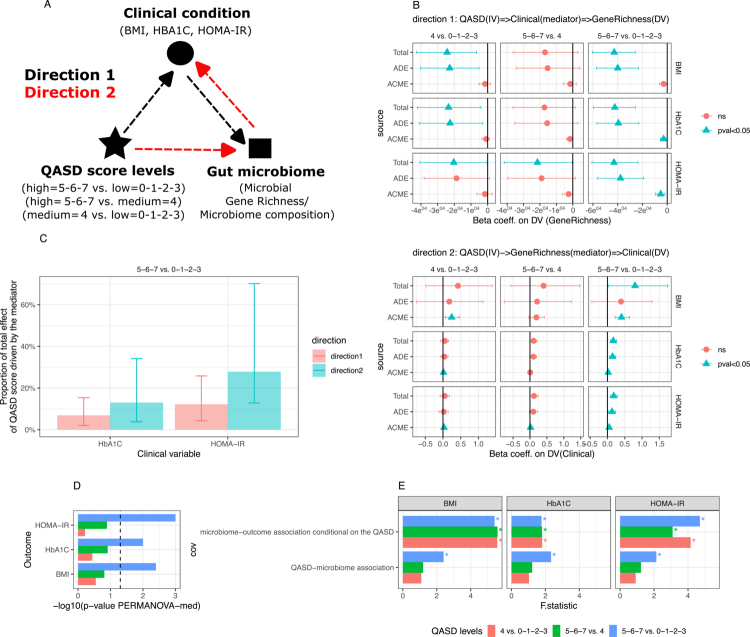
Mediation analyses of the impact of the QASD score on the gut microbiome and metabolic phenotypes. (A) Schematic representation of the two mediation hypotheses tested to evaluate the impact of the QASD score (independent variable) on the gut microbiome and clinical profiles of 1,643 individuals of MetaCardis study. In Direction 1, the QASD score impacts gut microbiome gene richness (GMGR; dependent variable), which is mediated by changes in clinical profiles (mediator). In Direction 2, the QASD score affects clinical profiles (dependent variable) mediated by alterations in the gut microbial gene richness (GMGR; mediator). The clinical variables tested included BMI, glycated hemoglobin, and HOMA-IR. Pairwise comparisons of the QASD score levels were conducted for low (0–1–2–3) vs. medium (4), low vs. high (5–6–7), and medium vs. high. Mediation analyses were adjusted for recruitment center, age, sex, and the use of metformin, statins, and PPIs (B) Confidence intervals of the beta coefficients representing the total effect (total), average direct effect (ADE), and average causal mediation effect (ACME) of the QASD score on the dependent variable in each direction (GMGR in Direction 1; clinical variables in Direction 2). Color and shape indicate the statistical significance of the mediation effects. Significant mediation in both directions (*p* < 0.05 for total, ADE, and ACME) was observed only between the upper and lower levels of the QASD score (high vs. low) with glycated hemoglobin and HOMA-IR. (C) Decomposition of the significant mediations observed between high and low levels of the QASD score, presented as a bar plot showing the proportion of the total effect and confidence intervals attributable to the mediator (clinical variable in Direction 1; GMGR in Direction 2). (D) Bar plot representing the -log10 transformed *P*-values (x-axis) in PERMANOVA-based mediation analyses of the mediation effect of the gut microbiome composition (Bray‒Curtis distances derived from MGS abundance profiles), with QASD score as the exposure and clinical variables as outcomes. The dashed line represents the nominal significance level (*p*-value = 0.05). (E) Barplots representing the F-statistic values (x-axis) for the exposure–microbiome association term and the microbiome–outcome association conditional on the exposure term in PERMANOVA-based mediation analyses (* = *P*-value < 0.05).

**Figure 3.**
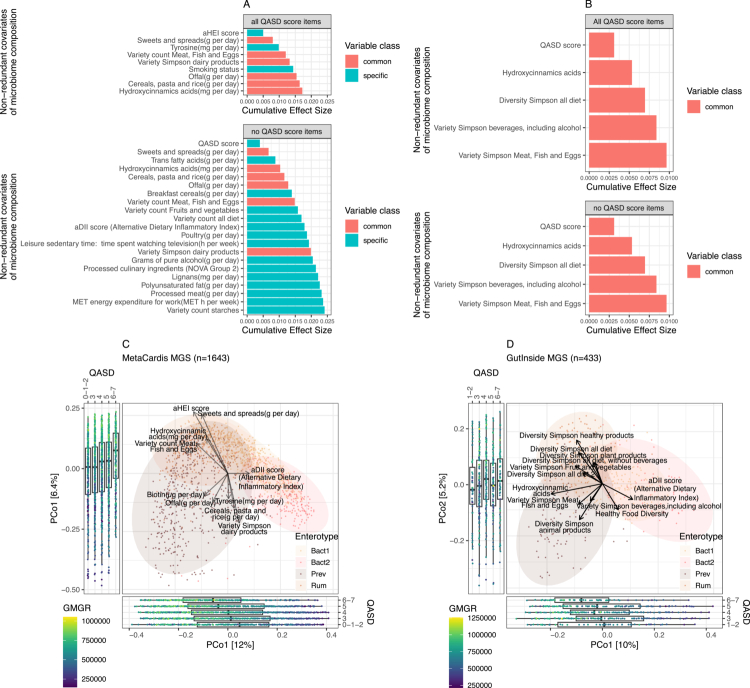
Links between lifestyle factors and microbiome composition in the MetaCardis and GutInside cohorts. (A) Bar plot showing the cumulative effect sizes of multivariate models for non-redundant microbiome compositional variation (adjusted cumulative R² values) in individuals from the MetaCardis cohort (*n* = 1,643). The results are presented for models including all individual components of the QASD score (“all QASD score items” panel; FDR < 0.01 in dbRDA analyses and *p* < 0.05 in stepwise model building, *n* = 9 variables) and for models excluding the four variables that define the QASD score (“no QASD score items” panel; FDR < 0.01 in dbRDA analyses and *p* < 0.05 in stepwise model building, *n* = 21 variables). (B) Bar plot showing cumulative effect sizes of multivariate models for non-redundant microbiome compositional variation (adjusted cumulative R² values) in individuals from the GutInside cohort (*n* = 433). The results are shown for models including all individual components of the QASD score (“all QASD score items” panel; *p* < 0.1 in dbRDA analyses and *p* < 0.05 in stepwise model building, *n* = 5 variables) and for models excluding the four QASD score variables (“no QASD score items” panel; *p* < 0.1 in dbRDA analyses and *p* < 0.05 in stepwise model building, *n* = 5 variables). (C) Principal coordinate analysis (PCoA) of inter-individual differences in microbiome profiles (based on Bray–Curtis dissimilarity from MGS abundance data) in the MetaCardis cohort (*n* = 1,643). The arrows in the main panel represent the effect sizes of a post hoc fit of 9 continuous nutritional covariates identified in the multivariate models from panel A. Boxplots represent the distribution of Metacardis individuals at different levels of the QASD score across 1^st^ and 2^nd^ ordination axis, with points coloured by GMGR. (D) Principal coordinate analysis (PCoA) of inter-individual differences in microbiome profiles (based on Bray–Curtis dissimilarity from MGS abundance data) in the GutInside cohort (*n* = 433). The arrows in the main panel represent effect sizes of a post hoc fit of 12 continuous nutritional covariates identified in the dbRDA analyses (*p* < 0.1). Boxplots represents the distribution of GutInside individuals at different levels of the QASD score across the 1^st^ and 2^nd^ ordination axis, with points coloured by GMGR. The full results from univariate and multivariate analyses are provided in Supplementary Tables S5 (MetaCardis data) and S6 (GutInside data). The common/specific legends in panels A and B corresponds to variables shared/unshared by models with/without the QASD score items.

**Figure 4.**
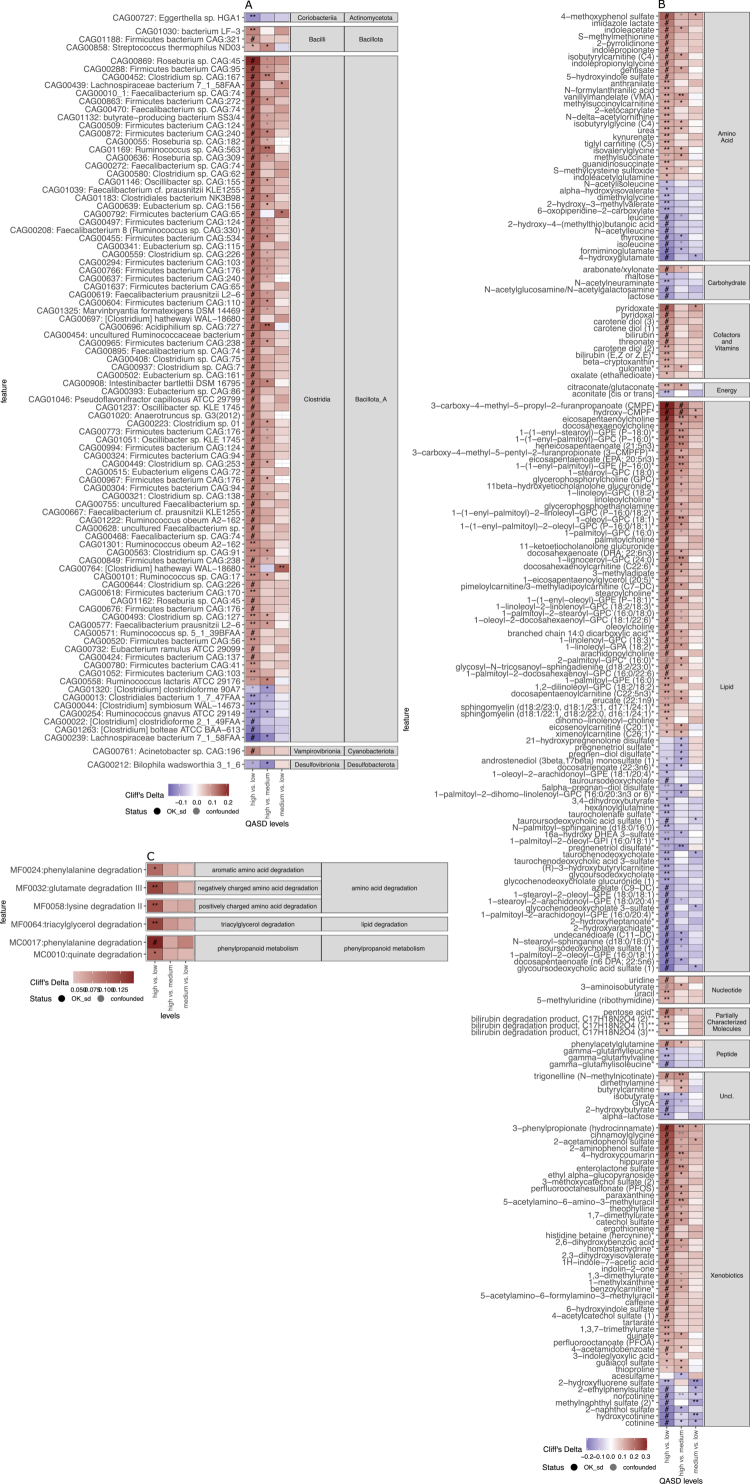
Microbiome and metabolome features associated with the QASD lifestyle score. (A) Heatmap representing the effect sizes (Cliff's delta) of 91 metagenomic species (MGS) showing significant differences between lifestyle score levels (high vs. low; high vs. medium; medium vs. low). Lifestyle score levels are categorized as low (QASD 0, 1, 2, 3), medium (4), and high (5, 6, 7). The effect sizes are strictly deconfounded by metformin, statin, PPI intake, and MetaCardis clinical groups, based on metadeconfoundR results (FDR < 0.1, OK_sd status meaning strictly deconfounded association, and absolute Cliff's delta > 0.1). Positive values indicate a higher abundance of the MGS at the reference level of the pairwise comparison (e.g., in HvsL, Cliff's delta > 0 denotes higher abundance at the “high” level of the lifestyle score compared to “low”, in red; Cliff's delta < 0 denotes lower abundance at the “high” level compared to “low”, in blue). The complete list of 122 MGS with significant differences between lifestyle score levels is available in Supplementary Table S8. (B) Same as panel A for 204 annotated serum metabolites with significant differences across lifestyle score levels (FDR < 0.1, OK_sd status, and absolute Cliff's delta > 0.1). The complete list of 319 metabolites with significant differences across lifestyle score levels is available in Supplementary Table S8. (C) Same as A-B for 6 gut metabolic modules (GMM) with significant differences across lifestyle score levels (FDR < 0.1, OK_sd status, and absolute Cliff's delta > 0.1). The text labels in heatmaps represent the significance level of the metadeconfoundR results (# = FDR < 0.001, ** = FDR < 0.01,* = FDR < 0.1).

**Figure 5.**
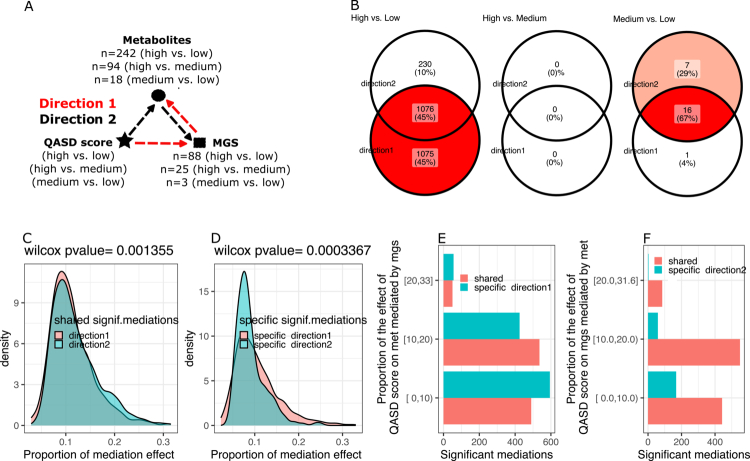
Summary of bidirectional mediation analyses between the QASD score, metabolites, and metagenomic species (MGS). (A) Schematic representation of the two tested directions for causal mediation driven by the QASD score (independent variable). In Direction 1, the QASD score affects serum metabolites (outcome variable) mediated by the abundance of MGS (mediator). In Direction 2, the QASD score impacts the MGS (outcome variable) mediated by serum metabolites (mediator). Mediation tests were conducted for all pairwise combinations of MGS and serum metabolites that showed significant variations between the QASD score levels in the drug deconfounded analyses. All the mediation analyses were adjusted for recruitment center, age, sex, and the use of metformin, statins, and PPIs. (B) Venn diagrams summarizing the number of significant mediations (FDR < 0.05 for average causal mediation effect (ACME), average direct effect (ADE), and total effect) found in each direction across pairwise QASD score levels. (C) Density plots showing the proportion of mediation effects in shared significant mediations for Direction 1 (MGS as a mediator; serum metabolites as outcomes) and Direction 2 (serum metabolites as mediators; MGS as outcomes). (D) Density plots illustrating the proportion of mediation effects in specific significant mediations for Direction 1 and Direction 2. (E) Decomposition of the 2151 significant mediation relationships in Direction 1 (QASD → MGS → metabolite) observed between high and low QASD score levels, based on the proportion of the total effect of the QASD score on serum metabolites mediated by MGS abundances (y-axis). (F) Decomposition of the 1306 significant mediation relationships in Direction 2 (QASD → Metabolite → MGS) observed between high and low QASD score levels based on the proportion of the total effect of the QASD score on MGS abundances mediated by serum metabolite levels (y-axis).

**Figure 6.**
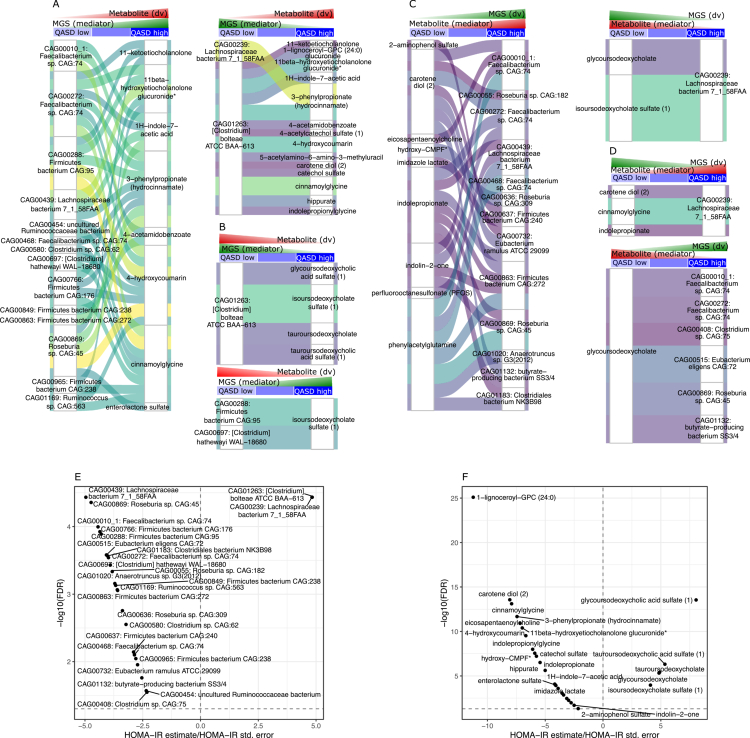
Overview of the strongest mediations between the QASD score-MGS-Serum metabolites in association with the HOMA-IR phenotype (A, B). Alluvial diagrams representing the 60 strongest significant mediation relationships (MGS-serum metabolites) in Direction 1 between high and low QASD score levels, categorized by the sign of the beta coefficients (increases/decreases in the mediator (MGS) and dependent variable (dv, serum metabolite) between high and low levels of the QASD score). (C, D) Alluvial diagrams representing the 41 strongest significant mediation relationships in Direction 2 between high and low QASD score levels, categorized by the sign of the beta coefficients (increases/decreases in the mediator (serum metabolite) and dependent variable (dv, MGS) between high and low levels of the QASD score). (E) Volcano plot representing the results of the linear regression analyses vs. HOMA-IR (dependent variable) of the 25 MGS included in the mediations represented in panels A–D. Dashed horizontal line represents the threshold for a significant association in multiple testing (FDR < 0.05 on 91 tested MGS). (F) Similar as E panel for the 28 serum metabolites included in mediations of panels A–D. Full results of mediation analysis and regressions vs. HOMA-IR are available in Supplementary Table S11.

The QASD score was computed by taking the unweighted sum of these four scores. The use of the unweighted sum of the subscores is justified because of its simplicity, neutrality, and robustness. In clinical settings, where scores based on variables often represent independent dimensions of patient status (e.g., physiological metrics, diagnostic findings, or treatment responses), the unweighted sum avoids introducing biases from arbitrary or uncertain weightings. This approach ensures equal contribution from each score, maintaining fairness when there is no clear evidence to prioritize one over the others, which is the case here. It also minimizes the risk of errors associated with miscalibrated weightings, which is critical in medical decision-making.^50^ Furthermore, the unweighted sum is transparent, easy to interpret, and widely applicable, making it appropriate for clinical contexts where clarity and comparability are essential. This straightforward aggregation method used to construct the QASD score aligns with evidence-based practices and is frequently used in composite medical indices and scoring systems.^51^ The QASD score ranges from 0 to 7. To address low numbers in some categories during stratification, we grouped the scores into five categories: “0–1–2,” “3,” “4,” “5,” and “6–7” for Figures 1, 3, and Supplementary Figures 2–5, and into three categories: “0–1–2–3,” “4,” and “5–6–7” for Figures 2, 4, 5, 6, and Supplementary Figures 6–8.

### Extraction of fecal genomic DNA and gut microbiota sequencing in MetaCardis cohort

Details of gut microbiota sequencing and taxonomic and functional profiling in the MetaCardis cohort has been extensively described in previous publications.^2^^,^^29^^,^^30^ Briefly, DNA from fecal samples was extracted following the International Human Microbiome Standards (IHMS) guidelines (SOP 07 V2 H). Shotgun sequencing was performed with ion-proton technology (Thermo Fisher Scientific; average 23.3 ± 4.0 million (mean ± SD) 150-bp single-end reads per sample). Alien Trimmer (v0.2.4)^52^ was used for quality filtering of raw sequencing reads, and non-bacterial contaminants of human and food origin were removed by aligning cleaned reads vs. human genome (RCh37-p10) and the genomes of *Bos taurus* and *Arabidopsis thaliana* (97% identity threshold). Filtered high-quality reads were aligned to the 9.9-million-gene catalogue of the human gut microbiome^53^using Bowtie2 (v.2.3.4; 95% identity threshold),^54^ from which a gene abundance table was obtained using METEOR v3.2 software^55^ (https://forgemia.inra.fr/metagenopolis/meteor). The read counts were downsized to 10 million reads per sample and normalized by the gene size and the number of total mapped reads reported in frequency using the R package MetaOMineR.^56^ From the downsized and normalized gene abundance table, functional and phylogenetic quantitative profiles were generated within the MOCAT2 framework.^57^ The abundance of 1435 metagenomic species (MGS; co-abundant gene groups with more than 500 genes corresponding to microbial species defined from 1,267 human gut metagenomes used to construct the 9.9-million-gene catalog as previously described^58^) were estimated as the mean abundance of the 50 genes defining a robust centroid of the cluster.^58^ The taxonomic annotation of the MGS was carried out by BLASTN of MGS genes against the NCBI database (November 2016 version), with species-level classification assigned when more than 50% of the genes matched the same reference genome with at least 95% sequence identity and 90% coverage. The GMGR was estimated from the average gene count across 10 rarefaction replicates.

### Extraction of fecal genomic DNA and gut microbiota sequencing in GutInside cohort

Gut microbiome sequencing of fecal samples from 433 individuals of the GutInside study^31^ was performed by GeneWiz (https://www.genewiz.com/) using Illumina HiSeq platform, resulting in 23.17 ± 5.1 million read pairs (mean ± std. deviation) per sample. Raw reads were processed with NGLess^59^ for quality trimming (minimum read quality = 25; minimum read length = 40), host contaminant removal vs. reference human genome (min. identity = 90%, min. match size = 45), alignment of filtered reads over the 9.9 million gene catalogue (min. identity = 95%, min. match size = 45), and generation of a gene abundance table with the *dist1* metric of NGLess, equivalent to the counting strategy described above for the MetaCardis cohort (first, the genome abundances are computed from unique mapped reads and then are corrected by the multiple mapped reads weighted by the coverage of unique mapped reads). The gene abundance table was processed for rarefaction and FPKM normalization with the MetaOMineR^56^ R package. The raw gene abundance table was rarefied to 10 million reads per sample, and the rarefied gene abundance table was normalized according to the FPKM strategy, from which MGS abundances were computed as in the MetaCardis cohort (mean abundance of the 50 genes defining a robust centroid of the cluster if more than 10% of these genes yielded positive signals). From the NGLess-filtered reads, genus-level abundance profiles in the mOTU space were generated with mOTU v2.6.1,^60^ from which enterotype classifications was performed after rarefy to 4000 counts per sample using the Dirichlet Multinomial Mixture (DMM) method^61^ and implemented in the Dirichlet Multinomial R package (Dirichlet-Multinomial Mixture Model Machine Learning for Microbiome Data. R package version 1.28.0). The minimum Laplace metric was used as criteria to select the best stratification of the cohort,^61^ which was at 4 discrete microbiome compositions that corresponded to the Rum, Prev, Bact1 and Bact2 enterotypes described in the Metacardis cohort.^62^

### Analyses of microbial diversity and community structure

The estimation of the explanatory power of nutritional features regarding relative microbiome profiles derived from MGS abundance data was performed using univariate and multi-variate stepwise distance-based redundancy analyses (dbRDA) as implemented in the R package *vegan* (Community Ecology Package. R package v.2.2-1).^63^ Redundance filtering of collinear lifestyle variables was performed prior to these analyses with the R package FMradio;^64^ threshold = 0.9 on the Spearman pairwise correlation matrix). The interindividual variation in the microbiome was visualized by principal coordinate analysis using Bray–Curtis dissimilarity on the basis of the relative abundance matrix derived from MGS abundance data. The environmental fit of nutritional covariates with a significant impact on microbiome composition based on dbRDA analyses over PCoA ordination from the Bray‒Curtis inter-sample dissimilarity matrix was computed with the *envfit* function of *vegan* R package. Associations of nutritional variables with enterotype status were tested with logistic regression analyses adjusted for age, gender, center, BMI, and the intake of metformin, statins and PPI.

### Metabolomic profiling

We used untargeted UPLC–MS data generated by Metabolon in serum samples and NMR data in urine samples. Full details of sample processing and generation of metabolomic profiles are available in our previous report.^30^

### Drug deconfounding analysis

The deconfounding pipeline was employed to determine the extent to which observed differences in microbiome and metabolome feature abundances between pairwise levels of the QASD score (high vs. medium, high vs. low, medium vs. low; low = QASD 1, 2, 3; medium = 4; high = 5, 6, 7) are influenced by confounding factors – such as treatment or risk variables – rather than being inherent characteristics of the specific phenotype itself. We used a post hoc filtering approach implemented in the R package *metadeconfoundR* (version 0.1.8; https://github.com/TillBirkner/metadeconfoundR or Zenodo), developed within the MetaCardis consortium.^29^ We followed the procedure described in.^30^ Medication status (statin, metformin, and PPI intake, all treated as binary variables) and the MetaCardis clinical group were considered as covariates, and the country of recruitment was included as a random effect. The analysis was conducted on metagenomic (MGS, mOTU, Gut Metabolic Modules (GMM)) and metabolomic features (serum, urine) present in at least 20% of the samples. For metagenomic features, abundances were corrected for bacterial cell count by multiplying by a normalization factor calculated as the bacterial cell count of the sample divided by the mean bacterial cell count across the entire dataset. Features with strictly deconfounded associations (SD), meaning that the QASD score adds significant explanatory power vs. a model with only the covariates (likelihood ratio test *p*-value < 0.05) and where the confidence interval does not include zero, were retained for further analyses.

### Statistical analyses

Data management and statistical analyses were performed using SAS version 9.3 and R (R statistical software, Vienna, Austria). The significance levels used were *p* <0.05, FDR <0.01, and *p* for interaction <0.1.

Several adjustment strategies were applied throughout the analyses. For analyses related to gut microbiome gene richness (GMGR), a normally distributed variable, we conducted sensitivity analyses considering potential confounders identified in previous MetaCardis consortium studies.^2^^,^^3^^,^^29^^,^^30^ These confounders included age, gender, recruitment center, antibiotic use, metformin and statin intake, and BMI. To isolate the independent effects of nutritional quality, the models were adjusted for energy intake (kcal) and the AHEI score, which is consistent with standard practices for dietary intake analysis. For specific omics features, we used the analysis pipeline developed and published in the MetaCardis study to ensure comparability with other studies (see the previous Drug Deconfounding Analysis section).

### Mediation analysis

Mediation analysis was used to infer causal mediation effects between metagenomic species (MGS), serum metabolites, and pairwise levels of the QASD score (high vs. low, high vs. medium, medium vs. low; low = QASD 1, 2, 3; medium = 4; High = 5, 6, 7). To reduce the number of tests, we focused on MGS and metabolites with strictly deconfounded status from the drug deconfounding pipeline, which exhibited an absolute effect size greater than 0.1 on Cliff’s delta between pairwise levels of the QASD score (as shown in Figure 4).

In the first step, pairwise associations between metabolites and MGS were tested individually using linear regression analyses, adjusted for country of origin, age, sex, and intake of metformin, statins, and proton pump inhibitors (PPI). Only significant pairs (FDR < 0.05) were retained for further mediation analysis. MGS abundances were normalized using the empirical normal quantile transformation method^65^ to ensure normality before analysis as proposed in.^66^

In the second step, we applied a similar regression framework, with the same adjustments, to identify significant mediations under two potential hypotheses regarding the direction of mediation, using the *mediate* function from the mediation R package (version 4.5.0).^67^ The first hypothesis examined the role of MGS as mediators in the relationship between the QASD score and the host's metabolomic profile (Direction 1, Figure 5A). The second hypothesis explored whether the relationship between the QASD score and the host metabolome (as a mediator) could subsequently influence the metagenomic landscape (Direction 2 in Figure 5A). Significant mediations were defined as those with an FDR < 0.05 for the average causal mediation effect (ACME), average direct effect (ADE), and total effect. Similar analyses were conducted to infer causal mediation effects between the levels of the QASD score, microbial gene richness, and clinical status of individuals, including measures of body mass (BMI) and metabolic status (glycated hemoglobin, HOMA-IR).

To further integrate insulin resistance profiles in the results of mediation analyses between the QASD, serum metabolites and MGS, we filtered significant mediations by retaining those where both the MGS and the serum metabolite were significantly associated with HOMA-IR in linear regression analyses under the same adjustment framework as in the mediation analyses (FDR < 0.05).

## Results

### Characterization of MetaCardis population with lifestyle variables

The 1643 studied individuals of the MetaCardis population include a metabolically healthy group (*n* = 305) and pathology groups defined as follows: individuals with metabolic syndrome (*n* = 220), type 2 diabetes (*n* = 477), obesity (BMI ≥ 30; *n* = 334), and cardiovascular diseases (*n* = 307) (Supplementary Table 1). Pharmacological treatments have been previously identified as factors that often mask disease-related signatures of the gut microbiome.^29^^,^^68^ Thus, medications such as 5-y retrospective exposure to antibiotics (total number of courses), metformin, statin and proton pump Inhibitors (PPI) usage were included as covariates in the analysis. Analyses of dietary variables were adjusted for total energy intake (in kcal/day), such as the AHEI score, while metagenomic analyses were adjusted for BMI or MetaCardis pathology groups (see Methods section and figure legends for details). To confirm some observations in independent populations, we used data from 433 participants of the GutInside study,^31^ which recruited French adults with overweight or obesity across French regions between September 2018 and January 2020. Baseline data were used from this dietary intervention program. Subjects’ phenotypes are provided in Supplementary Table 2. Both GutInside and MetaCardis cohorts used the same lifestyle assessment tools: e.g., Food Frequency Questionnaires (FFQ) for habitual dietary intake,^34^ the Recent Physical Activity Questionnaire (RPAQ) for physical activity and sedentary behavior^36^ and gathered smoking-related information, including past habits and current cigarette consumption. Shotgun metagenomic sequencing was available for both cohorts.^2^^,^^29^^,^^31^

### Dietary components, physical activity traits and smoke associate with gut microbiome gene richness

We first examined the relationships between the GMGR and individual variables, including food groups (*n* = 65), nutrients (*n* = 72), physical activity (*n* = 36) variables and smoking habits (*n* = 3, as qualitative variables). Among the 173 quantitative variables, 40 (23.12%) were significantly associated with the GMGR (FDR <  0.05; partial Spearman correlation analyses; Figure 1A). The adjusted effect sizes were modest (Spearman’s Rho ranging from −0.12 to 0.13) suggesting that lifestyle variables, considered independently, modestly impact GMGR variation. As shown in Figure 1A, among the food groups, positive associations with the GMGR were observed for the intake of alcoholic beverages (e.g., wine and beer), tea and coffee (classified as hot beverages), offal, nuts, and fish (including oily fish), as well as sweets and spreads. At the nutrient level, positive associations with the GMGR were found with alcohol, vitamins A/E, D, B7/B8 (e.g., biotin) and poly-unsaturated fatty acids and omega-6 fatty acids, as well as with the intake of total polyphenols and their subclasses, such as chalcones (found in beer), isoflavonoids (found in soja, onions, wine and tea) and hydroxycinnamic acids (found in coffee). These nutrient-based findings are consistent with those at the food group level and are in agreement with reports linking polyphenols to the gut microbiome diversity and health.^69^^,^^70^

Our analysis also revealed negative associations with the intake of trans fatty acids, previously implicated in diet-induced inflammation and containing bacteriostatic additives,^71^ and with sugars and carbohydrates. At the food group level, we observed a significant negative association between GMGR and “grains and starches” (rho = -0.08, FDR = 7.55e-03, Figure 1A), which was driven primarily by the intake of refined grains and simple sugars, which were grouped in this category. This food group shows the strongest negative association with the GMGR (e.g., “Refined grains and starches”; rho = −0.12, FDR = 1.15e-05; Figure 1A). This negative association was replicated in stratified analyses across the clinical subgroups (Supplementary Figure 2A). Conversely, a positive association between the intake of fruits and the GMGR was found in the healthy group (Supplementary Figure 2A; rho = 0.22, FDR = 0.016). With respect to physical activity, variables related to both occupational and leisure-time (partly reflecting total physical activity) were positively linked to GMGR (Figure 1A,B), while sedentary behaviors such as screen time in front of television or computer (indicator of sedentary behavior) were negatively associated with the GMGR (rho = -0.072, FDR = 0.018; Figure 1A). Smoking status was significantly associated with the GMGR, with current smokers showing a reduced GMGR (FDR = 3.72e-04; linear regression; Figure 1B). These associations remained significant after adjustments for clinical status and BMI (Supplementary Figure 2C,D).

Overall, we observed a majority of positive associations between the GMGR and many variables related to dietary intake, including some foods not typically considered healthy and physical activity, whereas sedentary behaviors, smoking, sugars and trans fatty acids were negatively associated with the GMGR. These observations are in agreement with prior studies in general populations,^7^^,^^72^ which also reported modest effect sizes for individual lifestyle variables.

### Dietary diversity and variety associate with microbiome gene richness regardless of dietary quality

To better capture the complexity of dietary behavior, we further explored associations between the GMGR and dietary scores, including those reflecting dietary diversity, variety and quality as well as the inflammatory potential of the diet. Considering commonly used dietary scores combining food items (e.g., AHEI, DASH, and the inflammatory score (DII)), the AHEI score, which reflects diet quality, showed a significant positive association with the GMGR (partial Spearman rho = 0.12, FDR = 1.28e-05; Figure 1C, adjusted model including age, recruitment center, energy intake (kcal/day), antibiotic treatments, and the use of metformin, statins, and PPI). In contrast, the aDII was negatively associated with the GMGR (partial Spearman rho = −0.07, FDR = 4.05e-03, Figure 1C, with the same adjusted model). No significant association was observed between the DASH score and the GMGR (Figure 1C). However, further analyses adjusting for diet quality (AHEI) revealed a nuanced picture: the previously significant association of the aDII with the GMGR became non-significant while the DASH score showed a significant negative association (partial Spearman rho = −0.07, FDR = 8.76e-03). These results likely reflect shared variance across dietary scores, as both DASH and aDII were positively or negatively strongly associated with AHEI (Spearman rho = 0.56, p value = 4.42e-136; Spearman rho = −0.44, *p* -value = 5.37e-81 respectively).

Other indices encompass diet diversity (“diversity all diet”), specific food group variety (“variety”), simple counts of different foods consumed within groups (“count”), and the evenness of food consumption within groups (“Simpson”). We therefore calculated 25 scores to assess these dietary patterns and examined their statistical relationships with the GMGR (see Methods and Supplementary Table S3 for detailed descriptions). We notably calculated the normalized composite “dietary and variety score of the diet”^46^^,^^47^ and the healthy food diversity index (HFD), which combines both diversity and variety dietary information.^45^ Fifteen of these scores were positively associated with the GMGR (Figure 1C; partial Spearman rho >0, FDR <0.05; fully adjusted model), including 7 of the 9 scores describing dietary diversity. Notably, 11 of these associations remained significant after adjustment for diet quality (assessed by the AHEI). Furthermore, for 2 scores (e.g., “Diversity Simpson all diet, without beverages” and “Variety count Meat, Fish and Eggs”), the associations with the GMGR were even strengthened after adjusting for AHEI (Figure 1C; fully adjusted model + AHEI; see Methods), with the “Diversity Simpson all diet, without beverages” score being the one with the highest increase in the strength of the association with the GMGR after adjusting for AHEI (Figure 1C; Rho ratio with/whithout AHEI, left panel).

By decomposing food diversity indices across food groups, we found that the “Variety count of whole grains and starches” had the strongest positive association with the GMGR, followed by the variety of “fruits and vegetables” (measured by the “count” index), “Beverages, including alcohol” (measured by the count and Simpson indices) and the Simpson index for “meat, fish, and eggs” (Figure 1C). Conversely, the variety count for “Refined grains and starches” showed a negative, though statistically non-significant, association with the GMGR. This finding aligns with the negative associations observed at the nutrient and food group levels for “sugars and simple starches”, as shown in Figure 1A. Importantly, the observed positive associations with the GMGR extended to diverse foods not typically considered healthy, such as a variety of “beverages, including alcohol”. The score “Diversity count all diet” was the score most strongly associated with the GMGR that remained associated after adjusting for dietary quality. In contrast to observations in other cohorts,^7^ the HFD score was not associated with GMGR in the MetaCardis population when considering the entire cohort or clinical subgroups (Figure 1C, Supplementary Figure 2B). Taken together, dietary diversity and variety, regardless of perceived food healthfulness, were positively associated with GMGR in this population with cardiometabolic disorders.

### A new composite lifestyle score is associated with GMGR, regardless of cardiometabolic phenotype severity

The modest effect sizes of individual nutritional variables and the influence of other lifestyle factors on GMGR variation in the MetaCardis population prompted us to develop an integrative metric. For this purpose, we built a composite lifestyle score, e.g., QASD for dietary quality, physical activity, smoking and dietary diversity, by assigning tertile points to diet quality (AHEI, 0/1/2), total physical activity (total physical activity in MET-h.week-1, 0/1/2), and the diet diversity score. We selected the “Diversity Simpson all diet, without beverages” score (0/1/2), because it was associated with GMGR that was statistically independent from AHEI (Figure 1C; full model + AHEI). Notably, the AHEI includes alcohol consumption, which was associated with the GMGR in univariate analysis. For smoking status, 0 point for current smokers and 1 for non-smokers were attributed. Using this additive approach by summing the scores for each component, participants received a total score between 0 (lowest QASD) and 7 (highest QASD), with higher QASD values reflecting more favorable lifestyle patterns. The distribution of QASD scores across the MetaCardis clinical groups showed significant differences (Figure 1D; chi-square test, *p* = 6.1e-09). The number of healthy individuals were significantly enriched in the high-QASD score levels (6–7; FDR < 0.05, post-hoc analysis for Pearson’s chi-square test for Count Data), while subjects with obesity were significantly overrepresented in the lowest-QASD score groups (0–1–2; FDR < 0.05, post–hoc analysis for Pearson’s chi-squared test for count data). In agreement, a higher QASD score was significantly associated with improved metabolic parameters, including lower levels of glycated hemoglobin, triglycerides and serum CRP, as well as better corpulence profiles (e.g., lower BMI, percent body fat and waist circumference; Supplementary Table S4; *p*-values < 0.05, Kruskal‒Wallis test).

Interestingly, we also observed significant differences in the distribution of QASD scores across the enterotypes defined in the MetaCardis population^2^ (Figure 1E, chi-square test, *p* = 1.3e-02), with the dysbiotic Bact2 enterotype being significantly enriched at low QASD score levels and significantly depleted at high QASD score levels (FDR < 0.05, post-hoc analysis for Pearson’s chi-squared test for count data). In line with this observation, we found a strong association between the composite QASD score and the GMGR in the whole population, which remained significant in several adjustment models that included the MetaCardis clinical groups as covariates (Figure 1F; FDR < 0.05; ANOVA test on linear regression models of the GMGR including the QASD and additional confounders; *p*-value < 0.05 on Tukey post-hoc pairwise tests across the QASD levels). The QASD score captured a broader range of GMGR variation than any of its individual components, notably the AHEI score (Figure 1F, Top Panel). This observation was partially replicated at the nominal *p*-value level in the GutInside cohort, where the QASD score outperformed its individual components in explaining GMGR variation (Figure 1G; *p*-value < 0.05; ANOVA test on linear regression models of the GMGR, including the QASD score and additional cofounders such as age and gender). Extending these analyses to other clinical variables in addition to the GMGR, such as BMI, HbA1c and HOMA-IR (as a proxy of insulin resistance), revealed significant associations between the QASD score with the three tested clinical variables across 8/9 regression frameworks, being particularly strong for HbA1c. Here, it consistently outperformed its individual components (Supplementary Figure 3A). Established scores such as the aHEI and physical activity were significantly associated with BMI and HOMA-IR in all adjustment models, with the aHEI showing the largest effect sizes for BMI and HOMA-IR (Supplementary Figure 3A). However, the QASD remained complementary, as it captured associations extending beyond single-behavior scores, particularly with HbA1c. These findings were not fully replicated in the GutInside cohort comprising only subjects with overweight and obesity, where only physical activity showed a significant association with HbA1C (Supplementary Figure 3B).

### Bi-directional mediation analysis reveals the QASD score's dual impact on the gut microbiome and metabolic health

The individual associations of the QASD score with the GMGR and with the clinical profile in the MetaCardis population led us to explore potential bidirectional mediation relationships between the QASD score, the gut microbiome, and clinical outcomes to better understand how these factors interact. We first evaluated the statistical interaction between the QASD score, GMGR and clinical variables considering routine and easily measurable phenotypes, e.g., body mass index (BMI) and glucose metabolic status (using glycated hemoglobin for glucose control and HOMA-IR), by conducting bi-directional mediation analyses under two different hypotheses with the QASD score as the independent variable (Figure 2A). The first hypothesis (direction 1 in Figure 2A) tested whether the QASD score influences the GMGR through variation in individuals’ clinical and biological variables (as mediators). The second hypothesis (direction 2 in Figure 2A) tested whether the QASD impacts individuals’ profile, via GMGR variation (e.g., as a mediator). We performed this analysis considering the upper and lower levels of the QASD score (QASD score 0–1–2–3; *n* = 662 vs. QASD score 5–6–7; *n* = 546) as well as each of these levels (e.g., “low” QASD or “high” QASD) versus intermediate QASD score level (QASD = 4; *n* = 435). The mediation analyses supported both hypotheses, especially when comparing upper and lower QASD score levels in relation to metabolic variables (high vs. low, Figure 2B, *P*-value < 0.05 for the total effect, average direct effect (ADE) and average causal mediation effect (ACME); mediation analyses adjusted by age, gender, recruitment center, and intake of metformin, statin and proton pump inhibitor).

In line with Hypothesis 1, variations in HOMA-IR and glycated hemoglobin explained 12.2% and 6.8%, respectively, of the influence of the QASD score on the GMGR (Figure 2C). However, our analysis also revealed that the QASD score significantly affects individuals’ glucose metabolism status and, that a significant proportion of this effect could be attributed to alterations in the GMGR. As such, in line with Hypothesis 2, 27.8% of the effect of the QASD score on HOMA-IR and 13.0% on glycated hemoglobin could be attributed to changes in the GMGR. Importantly, no significant mediation effect was observed when using BMI as an outcome or mediator. In additional support to these results, PERMANOVA-based mediation analyses^73^ using Bray-Curtis distances derived from gut microbiome composition confirmed the significant mediation effect of the gut microbiome on the association between lifestyle (QASD score) and clinical outcomes (BMI, HbA1C and HOMA-IR) when considering the upper and lower levels of the QASD score (Figure 2D–E).

Overall, we observed a significant bi-directional mediatory effect of GMGR and metabolic phenotypes on reciprocal effects of the QASD score on these variables, although with a stronger effect size when GMGR and gut microbiome composition is explored as mediator of QASD score effect on insulin resistance marker.

### The QASD score and specific dietary components explains variations in gut microbiome composition

Since GMGR represents only a facet of microbiome composition, we further extended our investigation to deeper evaluate the impact of QASD score together with other nutritional and lifestyle variable on gut microbiome composition both in MetaCardis population and in participants from the GutInside study for confirmation purpose. Distance-based redundancy analyses (dbRDA) identified 47 lifestyle variables significantly associated with microbiome composition, defined as the abundance of metagenomic species (MGS) pear individuals (Supplementary Table S5, FDR < 0.01). Among these, the AHEI score and the QASD score showed the greatest effect sizes, followed by the intake of sweets and spreads, “hydroxycinnamic acids” (e.g., coffee nutrient), “alcohol” and diet abundance of vitamins such as vitamin D, vitamin A and biotin. A significant association was also found between the inflammatory potential (aDII) of the diet and microbiome composition (Supplementary Table S5). Furthermore, stepwise forward model selection by permutation of these 47 variables provided a model comprising 9 variables with a non-redundant explanatory power for microbiome compositional variation which accounted for 1.72% of the total variance (Figure 3A). These variables included two components of the QASD score (e.g., “AHEI score” and “Smoking status”), the intake of “Sweets and spreads”, “Tyrosine, Offal”, “Cereals, pasta, rice” and “Hydroxycinnamic acids”, the “Variety count of Meat, Fish Eggs” and the “Variety Simpson dairy products” (Figure 3A, “all QASD score items” panel).

When the 4 individual variables composing the QASD score were excluded from the multivariate approach, the composite QASD score was the lifestyle variable with the highest non-redundant explanatory power in a multivariate model comprising 21 variables that explained 2.41% of the microbiome compositional variability (Figure 3A, “no QASD score items” panel). These findings highlight the added value of a composite such as the QASD score over its individual components in explaining microbiome variability in this population with cardiometabolic disorders. The multivariate model also included variables such as the inflammatory score of the diet and food variety scores and components (Figure 3A, “no QASD score items” panel). These observations were partially replicated in subjects with overweight and obesity from the GutInside study.^31^ Here, the QASD score exhibited the strongest association with microbiome compositional variation (adjR² = 3.11e-03, *p* = 0.019, Supplementary Table S6) and was retained as the variable with the highest non-redundant explanatory power, both with and without its individual components (Figure 3B). These models in the GutInside cohort shared variables with those derived from the MetaCardis cohort, including hydroxycinnamic acid intake and consumption of meat, fish, and eggs, captured by different diet diversity metrics (e.g., “Variety count” in MetaCardis, “Simpson diversity” in GutInside; Figure 3A,B). However, among the individual components of the lifestyle score, only the “dietary diversity excluding beverages score” (e.g., “Simpson Diversity Index for all diet, without beverages”) was significantly associated with dbRDA analyses in the GutInside cohort (adjR² = 1.14e-03, *p* = 0.048; Supplementary Table S6), although it was not retained in the final multivariate models shown in Figure 3B. Together, these findings underscore that while many individual dietary factors contribute modestly to gut microbiome variation, integrative metrics such as the QASD score may provide a more comprehensive assessment of microbial compositional differences across cardiometabolic populations.

### The QASD score associates to microbiome enterotype constellations

We next explored how the QASD score, related variables and other dietary components were associated with enterotypes. In previous studies, 4 distinct microbiome communities (e.g., enterotypes) were identified using Dirichlet Multinomial Mixture Models (DMM).^2^^,^^61^ These enterotypes correspond to partially overlapping ellipsoids in the PCoA ordination landscape based on Bray-Curtis beta-diversity distances (Figure 3C). Given the prominent dysbiotic character of one enterotype (e.g., Bacteroides 2 or Bact 2) in terms of microbiome diversity and microbial cell density, its strong association with obesity and inflammation^2^ and the significant differences in enterotype distribution observed across levels of the QASD score (Figure 1E), we evaluated in depth the association of enterotypes with the QASD score and nutritional/lifestyle variables with a significant impact on the gut microbiome composition. Projecting the 9 lifestyle variables (from Figure 3A) retained in the multivariate model onto this PCoA space revealed that the AHEI score, intake of “sweets and spreads”, “hydroxycinnamic acids” and the “variety count of meat, fish and eggs” were associated with the microbiome compositional space dominated by the Ruminococcus enterotype (Rum, Figure 3C). This enterotype has previously been associated with increased GMGR.^2^ A similar pattern was observed for additional variables retained in the multivariate model when the QASD components were excluded, e.g., breakfast cereals, lignans, polyunsaturated fats, dietary variety, starches, fruits and vegetables, and alcohol intake were positively associated with the Rum enterotype. Conversely, the intake of tyrosine, offal, “cereals, pasta and rice” and the variety of dairy products showed associations in the opposite direction in the ordination space (Figure 3C). This observation extended to trans fatty acids, poultry, processed meat and physical activity variables, such as leisure sedentary time and energy expenditure, which were associated with the opposing compositional direction.

Strikingly using our integrative metrics, QASD score levels showed a significant segregation along the 1 st and 2nd ordination axes of the PCoA (Figure 3C). The 1 st axis, which was strongly anticorrelated with GMGR (Spearman Rho = –0.6, *p* < 2.2 × 10^−16^), separated individuals with low QASD scores (0–1–2), who clustered at the extreme positive values of the axis corresponding to the Bact2 enterotype region. This enterotype was previously shown to be associated with a lower GMGR, a reduced bacterial load, systemic inflammation and obesity severity.^2^^,^^31^ In contrast, the second axis, strongly correlated with the GMGR (Spearman *ρ* = 0.7, *p* < 2.2 × 10^−16^), was enriched in individuals with high QASD scores (6–7) at its extreme positive values, corresponding to the Ruminococcus enterotype region (Figure 3C). These findings were partially confirmed in the GutInside population, where a high QASD score was associated with the Rum enterotype, while a higher aDII score was associated with the Bact2 enterotype, indicating moderate reproducibility of these associations in individuals with overweight and obesity (Figure 3D).

Logistic regression analyses of enterotype status versus the 47 nutritional covariates retrieved in dbRDA analyses in the MetaCardis population (adjusted for age, sex, BMI, center of recruitment and use of metformin, statins, and PPIs) confirmed the relationships between diet components and enterotype stratification. At the QASD score level, we found a significant decrease in the probability of belonging to the Bact2 group with increased QASD score levels, while no significant association was observed with Ruminococcus status (Supplementary Figure 4A). At the nutritional level, the “variety count of meat, fish and eggs”, the intake of offals, biotin, vitamin D (in food) and “cookies and pastries” were significantly associated with a decrease in the probability of belonging to the Bact2 enterotype (*p*-value < 0.05; Supplementary Figure 4B–F, Supplementary Results). This again emphasizes on the importance of dietary diversity and variety and not solely on food quality or the perceived healthiness of food. Interestingly, biotin intake in the diet was positively associated with the probability of the Prevotella enterotype in the logistic regression models (Supplementary Figure 4E, Supplementary Results), a community type dominated by bacteria that are auxotrophic for biotin production.^3^

Extended dbRDA analyses incorporating enterotype composition, clinical, biological and lifestyle variables confirmed that, despite the overlaps observed between enterotypes in the PCoA map, enterotypes largely outperformed other covariates in terms of explanatory power of gut microbiome compositional variation (Supplementary Figure 5A). Additionally, this analysis showed that in addition to BMI, fat mass, diabetes status and metformin intake, which explained the greatest variance, the AHEI and the QASD score were the most influential lifestyle variables on microbiome composition variance. The explanatory power of these lifestyle variables surpassed that of medications (e.g., statin intake) and biological variables such as lipid and hepatic blood markers (FDR < 0.01, dbRDA analyses; Supplementary Figure 5A, Supplementary Table S7). Other QASD components (such as smoking status, total physical activity without work (MET h week −1)) also emerged as significant contributors in these extended dbRDA analyses (FDR < 0.01; Supplementary Figure 5A). AHEI and smoking status were also retained in a multivariate model comprising 22 nutritional and clinical variables with non-redundant explanatory power, indicating that both the composite lifestyle score and clinical factors exert independent effects on microbiome compositional variation (Supplementary Figure 5B). Finally, associations between the QASD score and the gut microbiome composition were robust when adjusted for key confounders, including age, sex, country, BMI, metformin, statin and PPI intake (Supplementary Figure 5C). These findings highlight the value of considering composite lifestyle metrics, such as the QASD score, to capture relationships between dietary quality and diversity, lifestyle habits and the gut microbiome composition, including considered dysbiotic enterotypes such as Bact2.

### The QASD score associates with metagenomic species and blood metabolomic signatures

We next examined more in depth the relationships of the QASD score with metagenomic profiles (taxonomic and functional) and precision phenotypes evaluated by metabolomics. Given the heterogeneous profile of MetaCardis population in terms of clinical and medication profiles, we employed a post hoc filtering approach using the *metadeconfound* R package^29^ to account for the potential effects of medications (Metformin, statin, and PPI intake) and clinical status on putative associations of the QASD score with metagenomics and metabolomics measures. Among the examined features, MGS and serum metabolites presented the greatest number of significant and rigorously deconfounded associations, totaling 122 MGS and 319 serum metabolites with an FDR < 0.1. Most of these associations were statistically significant between the upper and lower levels of the QASD score (0–1–2–3, low-QASD, vs. 5–6–7, high-QASD) (Supplementary Figure 6). Only 6 and 11 features were retained in the functional modules (GMM, Figure 4C, discussed in the Supplementary Results) and mOTUs, respectively, while no urine metabolite was retained (Supplementary Figure 6; detailed results in Supplementary Table S8). We thus focused the subsequent analyses on 91 MGS and 272 serum metabolites with absolute effect sizes (Cliff’s delta) higher than 0.1.

At the MGS level, higher QASD scores were associated with an enrichment of *Firmicutes* lineages, including *Faecalibacterium prausnitzii* (12 MGS from the *Faecalibacterium* genus and 4 MGS corresponding to different *F. prausnitzii* strains, such as KLE1255 and L2-6), *Eubacterium eligens*, and multiple MGS from the *Oscillibacter* and *Roseburia* genera. In contrast, lower QASD scores were significantly linked to increased levels of *Clostridium bolteae* and *Ruminococcus gnavus* lineages (Figure 4A; Supplementary Results and Supplementary Table S9). We then examined which component of the QASD score captured the largest number of strictly deconfounded significant associations (e.g., using upper and lower levels of tertile decomposition for AHEI, physical activity and Simpson diversity index; smoking status as a binary variable). We found that AHEI and smoking status associate with 97 and 101 MGS, respectively whereas total physical activity and the Simpson diversity index were associated with fewer MGSs, e.g., 17 and 4 MGSs, respectively (Supplementary Figure 7A,B). This finding aligns with the significant, non-redundant impact of AHEI and smoking status on microbiome compositional variation (Figure 3A). Both smoking status and AHEI exhibited similar distributions of MGS changes when Cliff’s delta effect sizes were considered. This confirmed that most MGS showing strictly deconfounded associations were enriched in individuals with a high QASD score, high AHEI and non-smokers (Supplementary Figure 7C). Importantly, we also observed a significant positive association between the Cliff’s delta effect sizes of MGS changes with the QASD score and with its individual components, meaning that the overall QASD score captures the patterns of MGS variation in their individual components (Supplementary Figure 7D–G).

Regarding metabolomic signatures, a high QASD score was positively associated with hippurate, tryptophan-derived compounds, acyl-cholines, coffee-, and pyrimidine-related metabolites. Conversely, lower QASD scores were associated with elevated levels of branched-chain amino acids and their derivatives, bile acid metabolites, markers of tobacco consumption, and dipeptides (Figure 4B, Supplementary Results, and Supplementary Table S10). Deconfounding analyses of serum metabolites for the components of the QASD score showed results consistent with those described for MGS. AHEI and smoking status captured the greatest number of significant associations, with 311 and 377 serum metabolites, respectively, versus 103 and 70 for total physical activity and the Simpson diversity index (Supplementary Figure 7H–I). An increased fraction of specific metabolites was uniquely associated with either AHEI or smoking status (Supplementary Figure 7H–I). AHEI and the QASD score showed similar Cliff’s delta effect sizes, whereas smoking status showed a marked negative skew, reflecting enrichment of specific metabolites in smokers (Supplementary Figure 7J). These metabolites (Cliff’s delta < -0.5 for non-smokers vs. smokers; FDR < 0.1, strictly deconfounded status) included cotinine, hydroxycotinine and norcotinine, which are indicative of tobacco use. Notably, they were also identified through the QASD score, albeit with smaller effect sizes (Cliff’s delta ranging from −0.25 to −0.1; FDR < 0.1; Supplementary Figure 7J,N). Finally, we observed a significant positive association between the Cliff’s delta effect sizes of metabolite changes with the QASD score and with its individual components except the Simpson diversity index, indicating that the QASD score overall captures the patterns of metabolite variation in their individual components (Supplementary Figure 7K–N). Overall, this analysis identified distinct MGS and metabolic species associated with high versus low QASD scores, with the QASD score capturing a substantial proportion of metagenomic and serum metabolomic biomarkers of its individual components with comparable effect sizes.

### Gut microbiome strongly mediates the effect of the QASD score on serum metabolomics profiles

Finally, to integrate deconfounded individual MGS and serum metabolome biomarkers associated with the QASD score, we performed extended mediation analyses to assess tripartite relationships, testing whether the metagenome mediates the effect of the QASD score on serum metabolomic profiles, and conversely, whether serum metabolites mediate the impact of the QASD score on MGS abundances. We focused on 91 MGS and 272 metabolomic features that were robustly associated with the QASD score after a strict deconfounding approach (FDR < 0.1), and we demonstrated a substantial effect size (absolute Cliff’s delta effect size > 0.1). We tested two primary mediation hypotheses. The first hypothesis proposed that MGS mediates the relationship between the QASD score and the host's serum metabolome, indicating a microbiome-driven mediation pathway (Direction 1, Figure 5A). The second hypothesis explored an alternative scenario in which the host metabolome mediates the effect of the QASD score on the gut metagenomic landscape (Direction 2, Figure 5A). These hypotheses were tested across all deconfounded MGS-serum metabolite pairs stratified by the QASD score levels (high vs. low, high vs. medium, medium vs. low). importantly, for inclusion in this analysis, each MGS-serum metabolite pair exhibited significant reciprocal associations (FDR < 0.05, linear regression adjusted for center of recruitment, age, sex, and intake of metformin, statins, and PPIs).

Whereas bidirectionality was present, our findings strongly supported the preminence of the first hypothesis. Considering the high and low levels of the QASD score, there were 2,151 significant mediations in Direction 1 and 1,306 in Direction 2. Of these, 1,075 mediations (45.14% of the total) were specific to Direction 1, while 230 (9.65% of the total) were specific to Direction 2, and there were 1,076 significant mediations (45.19% of the total) in both directions (Figure 5B, Supplementary Table S11, Supplementary Figure 8). For intermediate QASD score levels, no significant mediations were observed for high vs. medium levels whereas only 40 significant mediations were found for medium vs. low levels, with 16 (40%) significant mediations in both directions (Figure 5B). This suggests that high and low levels of the QASD score have the most substantial impact on an individual's metagenomic and metabolomic profiles.

Focusing on high and low levels of the QASD score, we compared both directions in terms of the proportion of the mediation effect on the tested outcome variables. Significant mediations ranged from 2.28% to 34.03% of the total effect in Direction 1 (e.g., microbiome as a mediator) and from 3.89% to 31.5% in Direction 2 (metabolome as a mediator), revealing a significant difference between shared mediations despite overlapping distributions (*p* = 1.355e-03, Wilcoxon rank-sum test; Figure 5C). More importantly, when direction-specific mediations were examined, the mediation effect was significantly greater in Direction 1 (*p* = 3.367e-04, Wilcoxon rank-sum test; Figure 5D), indicating that both the number and strength of specific mediations are higher when the microbiome acts as the mediator.

This pattern was also observed when we stratified significant mediations by their strength in terms of the proportion of the mediating effect. We identified 106 and 88 high-impact mediations in each directions 1 and 2, respectively, where the mediator (MGS or serum metabolites, respectively) explained more than 20% of the effect of the QASD score on the exposure variable. Among these, 52.83% of the high-impact mediations in Direction 1 were specific (56/106), compared to 3.4% in Direction 2 (3/88). Similar trends were observed for medium-impact (10%–20%) and low-impact (<10%) mediations (Figure 5E–F).

Given the large number of significant mediations observed and considering the mediating role of the gut microbiome on the QASD-HOMA-IR relationship described earlier (Figure 2), we next focused on the detailed visualization of the strongest significant mediations where the MGS and the serum metabolite were both significantly associated with HOMA-IR (FDR < 0.05, linear regression analyses under the same adjustment framework as mediation analyses). Focusing on the strongest specific mediations in Direction 1 involving annotated serum metabolites (as exposures), we found 47 high-impact mediations (mediation effect > 20%) that involved both positive and negative relationships depending on the effect of the MGS on serum metabolite levels. This causal inference exploration reflects the complex network of interactions between gut microbiome species that may influence the insulin resistance-associated metabolome profile. The strongest mediations were observed in individuals with a high QASD score and involved serum metabolites such as cinnamoylglycine and 3-phenylpropionate, which were previously identified as biomarkers associated with high microbiome diversity.^74^^,^^75^ Higher levels of these metabolites in individuals with high QASD scores were strongly mediated by increases in several Firmicutes lineages, including *Faecalibacterium* and *Roseburia* (Figure 6A, left panel; Supplementary Table S11). Conversely, these same metabolites were also mediated by decreased levels of *Clostridium bolteae* (27.46% mediation for cinnamoylglycine) and *Lachnospiraceae bacterium 7_1_58FAA* (31.37% mediation for 3-phenylpropionate), two MGS enriched in individuals with low QASD scores (Figure 6A, right panel; Supplementary Table S11).

For individuals with low QASD scores, the strongest positive mediations involved increased levels of *Clostridium bolteae*, which significantly mediated the rise of four secondary bile acids, particularly isoursodeoxycholate sulfate (25.2% mediation effect; Figure 6B, top panel). This increase in isoursodeoxycholate sulfate was also mediated by decreased levels of two Firmicutes MGS (*CAG00697: [Clostridium] hathewayi WAL-18680*, 20.12% mediation effect; *CAG00288: Firmicutes bacterium CAG:95*, 22.85% mediation effect; Figure 6B, bottom panel; Supplementary Table S11).

In Direction 2, only 3 high-impact mediations (mediation effect > 20%) were observed. These included phenylacetylglutamine, whose rise in individuals with a high QASD score mediated the rise of *CAG00869: Roseburia sp. CAG:45* (20.99% mediation effect; Figure 6C, left panel), and the *CAG00239: Lachnospiraceae bacterium 7_1_58FAA*, whose decrease in individuals with a high QASD score was mediated by decreases in the secondary bile acid isoursodeoxycholate sulfate (24.95% mediation effect; Figure 6C, right panel) and increases in cinnamoylglycine (23.82% mediation effect; Figure 6D, top panel).

Extending the analyses to medium-impact mediations (mediation effect > 10%), mediations associated with high QASD scores included environmental exposures like food-derived carotenoids (carotene diol),^76^ which mediated increases in eight MGS (12.86% ± 1.74% mean ± standard deviation mediation effect), or Indolin-2-one, known for its antimicrobial activity,^77^ which mediated increases in four MGS (11.84% ± 1.1% mean ± standard deviation mediation effect), while perfluorooctanesulfonate, an environmental pollutant,^78^ mediated 12.37% of the increases in *CAG00439: Lachnospiraceae bacterium 7_1_58FAA* (Figure 6C, left panel). Additionally, metabolites derived from microbial metabolism, such as the aforementioned phenylacetylglutamine (a human metabolite synthesized from phenylalanine degradation),^79^ mediated increases in seven MGS (17.18% ± 2.58% mean ± standard deviation mediation effect), and indolepropionate (a tryptophan-derived metabolite)-mediated increases in eight MGS enriched in individuals with high QASD scores (11.51% ± 1.37% mean ± standard deviation mediation effect; Figure 6C, left panel, 6D, top panel). These findings suggest that microbially-derived serum metabolites may play a mediating role in shaping gut microbial communities across the upper and lower ends of the QASD score.

This mediating role was also observed with other secondary bile acids, such as glycoursodeoxycholate, which, in individuals with low QASD scores, mediated increases in *CAG00239: Lachnospiraceae bacterium 7_1_58FAA* (13.33% mediation effect, Figure 6C, right panel) and decreases in six Firmicutes lineages (13.07% ± 2.21% mean ± standard deviation mediation effect; Figure 6D, bottom panel, Supplementary Table S11).

In addition, although the MGS and serum metabolites involved in the mediations described above were all significantly associated with HOMA-IR, we also identified significant mediations driven by environmental exposures such as tobacco consumption. For example, cotinine, enriched in individuals with low QASD scores, significantly mediated decreases in several Firmicutes lineages (e.g., CAG00055: *Roseburia sp. CAG:182* and CAG00010_1: *Faecalibacterium sp. CAG:74*), despite cotinine itself not being significantly associated with HOMA-IR (mediation effects of 14.08% and 20.51%, respectively; Supplementary Table S11).

Finally, when we examined the linear regression results for the MGS and serum metabolites involved in these mediations, we found a clear pattern: secondary bile acids and MGS that were increased in individuals with low QASD scores (such as CAG01263: *[Clostridium] bolteae ATCC BAA-613* and CAG00239: *Lachnospiraceae bacterium 7_1_58FAA*) were positively associated with HOMA-IR. In contrast, other serum metabolites and MGS that were increased in individuals with high QASD scores were negatively associated with HOMA-IR (Figure 6E,F).

These findings highlight a predominant microbiome-to-metabolome mediation axis, particularly involving Firmicutes lineages, drived by the nutritional and lifestyle factors summarized in the QASD score, that could shape insulin resistance–associated profiles. While bidirectionality exists, the mediating effects were stronger when the microbiome acted as a mediator, and strong mediatory links, such as the rise of secondary bile acids, emerged in individuals with low QASD scores mediated by *CAG01263: [Clostridium] bolteae ATCC BAA-613.* These links underscore the ability of the QASD score to capture nutritional and environmental signatures that impact host phenotypes through alterations in the gut microbiome composition with links to the insulin resistance profiles of the host.

## Discussion

We herein investigated how integrated lifestyle behaviors, captured by a composite lifestyle score (QASD), are related to gut microbiome gene richness and composition (including enterotype stratification) and host metabolic phenotypes in healthy individuals and those with cardiometabolic disorders. Whereas bidirectional interactions are evident, our findings highlight a significant mediating role for the GMGR in the association between QASD and glucose metabolism markers (HbAIc and HOMA-IR). By applying extended causal mediation analyses, we confirmed that the gut microbiome is a significant mediator in the impact of the QASD score on host phenotypes in terms of insulin resistance as well as serum metabolomic profiles. Accordingly, by accounting for different directions in mediation analyses, we identified interplays between insulin resistance-associated bacterial species and serum metabolites, with putative beneficial or detrimental effects on cardiometabolic health.

We first observed that GMGR is not only associated with a large variety of individual food items, macronutrients and micronutrients including polyphenols and vitamins, but also with broader dietary patterns, measures of physical activity and smoking behavior. On the dietary side, we showed that associations between GMGR and dietary diversity (evaluated by different metrics) can be independent of dietary quality. Previous observations in healthy populations such as PREDICT and Flemish Gut cohorts,^6^^,^^7^ also showed that dietary diversity is associated with the GMGR. Our results are also in agreement with recent findings that omnivorous diets – more diverse than vegetarian or vegan diets – are associated with higher microbial diversity.^15^ Moreover, our identified positive associations between physical activity items and the GMGR were in line with observations made in healthier populations recruited in the US Arrivale cohort.^80^ Overall, our findings demonstrate that, in individuals with a spectrum of cardiometabolic disorders, greater GMGR is significantly associated with increased dietary diversity, even when this diversity includes less healthy food items such as alcohol. The polyphenolic compounds present in alcoholic beverages such as red wine could contribute to these associations, as shown in previous studies.^81^ We should also consider that patients included in the MetaCardis study reported globally moderate alcohol consumption, with high alcohol consumption being a criteria of exclusion.

Finally, even if high GMGR is generally considered a proxy for gut health, this is not the case in certain contexts, where high microbiome diversity could be associated with non-beneficial exposures.^82^^,^^83^ Our results underscore the clinical importance of distinguishing between the diversity and quality of food consumption, as greater food variety, if generally recommended, does not inherently reflect healthy eating patterns or more favorable microbiome configurations in populations with pathologies.

Our observations extend beyond the GMGR. Combining lifestyle variables most strongly associated with the GMGR, which include, for example, alcohol as part of the AHEI, demonstrated a greater impact on microbiome composition across the MetaCardis population and pathology subgroups, compared to its individual components. Nevertheless, the effect size of individual dietary or lifestyle factors on gut microbiome compositional variation remains limited, a trend similarly observed in many other populations^84-86^. The four QASD components (e.g., dietary quality and diversity, physical activity levels and smoking status) appear to exert partially non-redundant effects. When we examined the most influential lifestyle variables that had non-redundant associations with microbiome composition, we found that they collectively accounted for less than 1.8% of the microbiome's variance. When clinical covariates were included, the explained variance moderately increased (approximately 2.5%), with AHEI and smoking status identified as key non-redundant contributors. These findings highlight, in individuals with cardiometabolic diseases, the multifactorial nature of microbiome variation shaped by complex interactions among lifestyle behaviors, diet, clinical status, and potentially genetic and other environmental influences not captured in this analysis.^6^^,^^87^^,^^88^

We described that a low QASD and a high dietary inflammatory index (aDII) were linked to the Bact2 enterotype both in MetaCardis and the GutInside validation cohort. While the concept of enterotypes, which was originally defined based on beta-diversity, has been subject to debate since its introduction,^61^^,^^89^^,^^90^ a growing scientific consensus recognizes that enterotypes represent compositional archetypes within a continuous gradient of gut microbiome community structures.^91^ Specific bacterial constellation, such as the Bacteroides-dominated, low-diversity Bact2 enterotype is of particular interest since it has been linked to higher BMI and elevated inflammatory markers in different cohorts, including MetaCardis.^2^^,^^31^ Here, a combination of diet items, patterns and lifestyle features explained some of these associations in the multivariate models. Interestingly, the association between increased intake of specific foods, such as offals, biotin- and vitamin D-rich items, and even cookies and pastries, and reduced Bact2 prevalence suggests that dietary diversity may help modulate dysbiotic microbiome profiles. In line with this hypothesis, in mouse experiments, in the context of diet-induced obesity, that the combined supplementation of biotin with fructooligosacharide leads to a significant improvement of metabolic health phenotype and gut microbiome diversity.^3^ Our observational findings should nevertheless be interpreted with caution in humans, as most dietary components account for a small fraction of microbiome variance. This underscores the need for tailored, multi-component interventions to evaluate their clinical impact and determine whether putative effects are mediated through microbiome-related mechanisms.

We further explored the metagenomic and host metabolomic signatures and found significant associations between the QASD score and 91 identified MGSs and 272 serum metabolites. Many of the QASD-based associations are consistent with previously reported health-status associations (discussed in depth in the Supplementary Results and Supplementary Tables 9 and 10). We found that MGS enriched in individuals with high QASD scores belonged to bacterial groups such as *F. prausnitzii, E. eligens, Oscillibacter*, or *Roseburia* lineages previously described with health-promoting effects and found to be depleted in inflammatory diseases,^7^ emphasizing the contribution of lifestyle patterns to these bacterial modulations. Conversely, lower QASD scores were associated with MGS previously linked to systemic and postprandial inflammation and deteriorated health, such as *C. bolteae* and *R. gnavus.*^7^ The QASD score captured metabolomic signatures of clinical interest, including higher levels of microbiota- and diet-derived metabolites such as hippurate, acyl-cholines, and tryptophan-related compounds, while lower QASD scores were associated with branched-chain amino acids and tobacco-related metabolites. Given the relevant associations with microbial species and metabolites consistently linked to health-promoting or disease-associated states, these results stimulated explorations of the complex interplay between the QASD score, gut microbiome composition, and metabolic phenotypes.

Interventional studies on personalized diets have indeed demonstrated that gut microbial pathways and species, including subgroups of *Faecalibacterium prausnitzii*, can mediate the effects of various dietary components on metabolic health.^92^^,^^93^ Research from the LifeLinesDEEP cohort highlighted the mediating role of microbial vitamin B1 and B2 production in the relationship between fruit intake and diabetes risk.^94^^,^^95^ Findings indicate that the gut microbiome modulates the beneficial association between adherence to a Mediterranean diet and cardiometabolic risk, using prospective data from 307 participants.^96^ Moreover, microbiome diversity and composition were also described as mediators of the beneficial effects of a green Mediterranean diet on reducing cardiometabolic risk.^97^

Our mediation analyses, in this cross-sectional design, demonstrated that interactions between the gut microbiome and host metabolism are bidirectional but strongly support a prominent pathway in which lifestyle factors influence the microbiome, which in turn mediates effects on metabolic phenotypes. First, the GMGR significantly mediated the association between the QASD score and clinically relevant glucose metabolism markers, e.g., HbAIC and HOMAIR, while BMI showed no significant mediation relationship. Clinical variables linked to glucose metabolism also mediated the link between lifestyle and the GMGR but with lower mediation effects. These results align with previous evidence that the gut microbiome modulates host glucose homeostasis rather than adiposity.^98^

Our larger-scale mediation analyses expand upon this initial observation linking the GMGR to routine clinical markers by demonstrating, at a systems level, that the gut microbiome significantly mediates the effects of lifestyle on host metabolomic profiles. This was striking in the QASD-associated MGS–metabolite pairs, where the microbiome-to-metabolome direction yielded both a higher number of significant mediations overall (2,151 vs. 1,305 mediations) and direction-specific (1075 vs. 230 mediations) together with larger effect sizes compared to the reverse direction. These results provide strong evidence that specific gut microbial species play pivotal and eventually directional role in translating lifestyle patterns into systemic metabolomic phenotypes. Actually, *Faecalibacterium* and *Roseburia* lineages (e.g., associated with high QASD), were key microbial mediators, found to mediate increases in blood metabolites such as cinnamoylglycine and 3-phenylpropionate, both biomarkers of high microbiome diversity listed in the Microbial Metabolome Database (MiMeDB).^99^ Cinnamoylglycine, in particular, has been shown to have a microbial origin in mouse studies, suggesting a need for future mechanistic research.^100^ Conversely, *Clostridium bolteae* emerged as a key mediator of increased secondary bile acids, such as isoursodeoxycholate sulfate, in individuals with low QASD scores. This serum biomarker has been linked to postprandial lipemia, inflammatory diseases and impaired liver function.^101^ In support of a mechanistic interpretation, genome-scale metabolic models of *C. bolteae* from the AGORA2 repository (including the ATCC_BAA_613 strain) contain bile salt hydrolases that deconjugate primary bile acids, producing the isoursodeoxycholate precursor ursodeoxycholate.^102^^,^^103^ In contrast, we found that serum metabolites reflecting environmental exposures, such as cotinine (a marker of tobacco consumption) or carotene diol (food-derived carotenoids), significantly mediated the impact of the QASD score on gut microbial species. This mediation involved metabolites of microbial origin, including indolepropionate (from microbial degradation of dietary tryptophan), phenylacetylglutamine (from dietary phenylalanine), and secondary bile acids such as glycoursodeoxycholate and isoursodeoxycholate sulfate. These findings reinforce the fact that gut microbiome activity is modulated by metabolic responses captured through the serum metabolome, again supporting the concept of bidirectional mediation. Importantly, MGS and serum metabolites highlighted in these mediations were significantly associated with insulin resistance, revealing a robust enrichment pattern of secondary bile acids in individuals with low QASD scores and high HOMA-IR. This was particularly evident for *CAG01263: [Clostridium] bolteae ATCC BAA-613,* which mediated the abundance of secondary bile acids, and *CAG00239: Lachnospiraceae bacterium 7_1_58FAA* also linked to metabolites associated with insulin resistance. Notably, the enrichment of secondary bile acids in individuals with insulin resistance and T2D has recently been reported in two Swedish cohorts,^104^ further supporting these findings in the MetaCardis population.

This study has several limitations, including its cross-sectional design and reliance on FFQs to assess long-term dietary patterns, which may overlook short-term intake variability. Future studies should consider incorporating 24-h recalls to capture short-term dynamic dietary fluctuations. The QASD score, while useful as a composite index reflecting overall lifestyle quality, may mask specific mediation effects driven by individual nutrients – such as tryptophan or phenylalanine – on metabolite levels via distinct microbial species. Previous work has shown both microbiome-mediated effects of diet on metabolites (e.g., *Ruminococcus*-mediated impact of fruit intake on urolithin B) and reverse pathways (e.g., plasma hippurate mediates the effect of coffee on *Methanobrevibacter smithii*). ^87^

The statistical strength of the associations of the QASD score with the GMGR and gut microbiome composition observed in the MetaCardis population decreases in our validation cohort (GutInside). This could be explained by differences in cohort characteristics: GutInside is a more homogeneous population (subjects with overweight and moderate obesity from France) compared with MetaCardis, which spans a broader range of geographic origins and cardiometabolic conditions. The smaller sample size of GutInside (*n* = 433 vs. *n* = 1643 in MetaCardis) reduces the statistical power to detect associations of similar magnitude. Because the QASD score is derived from population-specific tertile distributions, part of the differences observed between cohorts (e.g., MetaCardis vs. GutInside) may reflect underlying variation in lifestyle component distributions. In this context, MetaCardis could serve as a reference population to calibrate QASD score categories in smaller or less diverse cohorts, but the reduced association in the GutInside population also highlights the importance of cohort context when interpreting lifestyle–microbiome associations and underlines the need for future studies in larger and more diverse populations. Finally, it remains challenging to fully disentangle whether the observed associations between QASD and microbial species or blood metabolites are direct, or instead secondary to gut microbial gene richness (GMGR) or host health status. These relationships are inherently complex, involving multiple potential interaction pathways, which may confound causal inference. In this context, while mediation analysis is a powerful approach for exploring pathways linking lifestyle, microbiota, and metabolism, it inevitably simplifies the complex, bidirectional nature of these interactions and cannot establish definitive causality.

In conclusion, our study highlights the complex and bidirectional influence of lifestyle factors on the gut microbiome and host metabolic phenotype in individuals with cardiometabolic disorders. Through causal mediation analysis, we identified insulin resistance-associated microbial taxa and metabolites, such as *Faecalibacterium prausnitzii, Clostridium bolteae*, secondary bile acids, and indolepropionate, as key mediators linking the composite lifestyle score (QASD) to both host metabolomic profiles and the gut microbiome composition. These findings pave the way not only for mechanistic pathway exploration but also for potential biomarkers for identifying at-risk individuals and tailoring interventions. We propose the QASD score as an integrative composite tool to assess lifestyle–microbiome–host interactions and guide personalized nutritional or behavioral strategies. Ultimately, the proposed mediation framework offers a path toward prevention and monitoring of metabolic health in lifestyle intervention.

**MetaCardis consortium collaborators**

The MetaCardis consortium †: Renato Alves, Ehm Astrid Andersson Galijatovic, Olivier Barthelemy, Jean-Philippe Bastard, Jean-Paul Batisse, Randa Bittar, Matthias Blüher, Frederic Bosquet, Olivier Bourron, Mickael Camus, Cecile Ciangura, Arne Dietrich, Morad Djebbar, Angélique Doré, Line Engelbrechtsen, Leopold Fezeu, Sebastien Fromentin, Nathalie Galleron, Marianne Graine, Caroline Grünemann, Agnes Hartemann, Bolette Hartmann, Gerard Helft, Malene Hornbak, Charlotte Kranepuhl, Julien Chilloux, Antonis Myridakis, Lesley Hoyles, Richard Isnard, Sophie Jaqueminet, Niklas Rye Jørgensen, Hanna Julienne, Johanne Justesen, Judith Kammer, Nikolaj Karup, Lars Køber, Michael Kuhn, Véronique Lejard, Ivica Letunic, Florence Levenez, Lajos Marko, Laura Martinez-Gili, Nicolas Maziers, Lucas Moitinho-Silva, Gilles Montalescot, Ana Luisa Neves, Michael Olanipekun, Laetitia Pasero Le Pavin, Luis Pedro Coelho, Nicolas Pons, Benoit Quinquis, Andrea Rodriguez-Martinez, Hugo Roume, Sebastien Schmidt, Tatjana Schütz, Johanne Silvain, Mathilde Svendstrup, Thierry Vanduyvenboden, Stefanie Walther.

## Supplementary Material

Supplementary material Reviewed_Manuscript_Nutrition_18112025_SupplResults.docx

Supplementary material Descriptive_tables_18112025

## Data Availability

Raw shotgun sequencing data that support the findings of this study have been deposited in the European Nucleotide Archive with accession codes https://www.ebi.ac.uk/ena/browser/view/PRJEB37249, https://www.ebi.ac.uk/ena/browser/view/PRJEB38742, https://www.ebi.ac.uk/ena/browser/view/PRJEB41311, https://www.ebi.ac.uk/ena/browser/view/PRJEB46098 and https://www.ebi.ac.uk/ena/browser/view/PRJEB71898, with public access. Metabolome data have been uploaded to MassIVE with accession numbers MSV000088042 (serum GCMS) and MSV000088043 (additional isotopically 97 quantified serum metabolites using UPLC–MS/MS). In adherence to EU and national privacy laws, unrestricted access to patient phenotypic data cannot be provided for MetaCardis. Researchers wishing to access individual phenotypic data would need to submit argued applications to the relevant National Data Protection Agencies. These are the Danish Data Protection Agency (https://www.datatilsynet.dk/english) for phenotypic data from study participants recruited in Denmark, the Federal Commissioner for Data Protection (https://www.bfdi.bund.de/EN/Home/home_node.html) for phenotypic data from study participants recruited in Germany and the Commission Nationale Informatique & Libertés (https://www.cnil.fr/en/home) for phenotypic data of study participants recruited in France. The application procedures are described on the outlined websites. If such permission is granted, phenotypic data will be then made available by the corresponding authors within 5 weeks.
