# Supplementary material for: Prominent mediatory role of gut microbiome in the effect of lifestyle on host metabolic phenotypes
Source: Gut Microbes. 2025 Dec 17;17(1):2599565. doi: 10.1080/19490976.2025.2599565 (PMC12795280; doi:10.1080/19490976.2025.2599565)
Supplement: Supplementary material — Reviewed_Manuscript_Nutrition_18112025_SupplResults.docx [file KGMI_A_2599565_SM3719.docx]

Supplementary information of manuscript *Prominent mediatory role of gut microbiome in the effect of lifestyle on host metabolic phenotypes*

[Supplementary Figures 1](#_Toc200535373)

[Supplementary Results 9](#_Toc200535374)

# Supplementary Figures

**Suppl. Figure 1: Flowchart of study design based on Metacardis population**

**Supplementary Figure 2: Sensitivity Analysis of Dietary and Physical Activity Factors, Diet Variety, and Diversity Scores Associated with Gut Microbiota Gene Richness (GMGR) in the MetaCardis Population** **(A)** Heatmap showing the coefficients of partial Spearman correlations between GMGR and various nutritional and lifestyle factors (y-axis) across the entire MetaCardis cohort (all levels on the x-axis, corresponding to results in Figure 1A) and within individual clinical subgroups (additional x-axis levels). **(B)** Heatmap displaying the coefficients of partial Spearman correlations between GMGR and dietary indices (y-axis) in the full MetaCardis cohort (all levels on the x-axis, corresponding to results in Figure 1C) and across clinical subgroups (additional x-axis levels). Symbols indicate significance: # = FDR < 0.05; * = p < 0.05, FDR > 0.05. Correlations are adjusted for age, recruitment center, energy intake (kcal), antibiotic treatments (number in the past five years), and use of metformin, statins, and PPIs. **(C)** Estimated marginal means of GMGR across tertile levels of total physical activity, as well as different levels of smoking status, derived from linear regression models adjusted for age, recruitment center, energy intake (kcal), antibiotic treatments, use of metformin, statins, PPIs, and BMI. **(D)** Same as panel (C) but additionally adjusted for clinical group. Symbols denote significance: ** = FDR < 0.05; * = p < 0.05, FDR > 0.05, based on ANOVA tests on linear regression models.

**Supplementary Figure 3: Extended associations of QASD score and its individual components with BMI, HbA1C and HOMA-IR in MetaCardis (panel A) and GutInside (Panel B) cohorts.** Top barplots represents the effect sizes (F-values) derived from Anova Type III test on linear regression models where each clinical variable (dependent variable; top vertical facet) is regressed against the QASD score and its individual components (y-axis, 1 regression model x independent variable) under different adjustment frameworks (Ref. adjustment: age + recruitment center + energy intake (kcal) + antibiotic treatments, and use of metformin + statins + PPIs; group=MetaCardis clinical groups). Bottom panels represent the estimated marginal means of each clinical variable across QASD score levels and quintiles of the distribution of QASD score individual components derived from the same linear regression models (**: FDR<0.05, *=P-value<0.05 & FDR>0.05, NS=Non-significant; Anova Type III test).

**Supplementary Figure 4: Nutritional Variables Significantly Associated with Enterotype Status in the MetaCardis Cohort**

Logistic regression models were fitted for each enterotype (*Bacteroides 2, Bacteroides 1, Ruminococcus,* and *Prevotella*) against 47 nutritional variables identified in dbRDA analyses, adjusted for age, sex, BMI, recruitment center, and use of metformin, statins, and PPIs. Eight variables showed significant associations with at least one enterotype, represented as predicted probabilities based on the logistic regression models (*=*p* < 0.05 on logistic regression of target enterotype status (0,1) vs. nutritional variable). (**A**) Higher QASD score levels (5, 6-7) were associated with a significantly lower probability of the *Bacteroides 2* (Bact2) enterotype. Additionally, Bact2 status was negatively associated with five other nutritional variables (**B-F**), including “Variety count of Meat, Fish, and Eggs,” intake of various vitamins (biotin, vitamin D), consumption of “Cookies and Pastries,” and offal intake—where higher values of these variables were significantly linked to a lower probability of being in the Bact2 enterotype. (**G-H**) The *Prevotella* enterotype was positively associated with the intake of dairy products (Simpson diversity of dairy products) and vitamin A, as well as with offal consumption (**D**) and biotin intake (**E**), which were also associated with a decreased probability of the Bact2 status.

**Supplementary Figure 5: Impact of Enterotypes, Nutritional and Bioclinical Variables on Microbiome Composition in the MetaCardis Cohort**

**(A)** Bar plot showing the effect sizes of enterotype stratification and 73 covariates (nutritional and clinical) with a significant impact on microbiome composition in individuals from the MetaCardis cohort (n = 1,643), as determined by distance-based redundancy analysis (dbRDA) using Bray-Curtis dissimilarity from MGS abundance data (FDR < 0.01). **(B)** Bar plot of the 30 clinical and nutritional covariates with the highest significant impact on microbiome composition in the MetaCardis cohort (n = 1,643). Effect sizes are presented either independently (univariate effect sizes in black; FDR < 0.01 in dbRDA) or within a multivariate model (cumulative effect sizes in gray). The red line indicates the cut-off for significant non-redundant contribution to the multivariate model (p < 0.05 in stepwise model building). Full results are provided in Supplementary Table S7. **(C)** Bar plot representing the effect sizes of the QASD score on microbiome composition (n = 1,643) using different adjustment frameworks, as assessed by distance-based redundancy analysis (dbRDA) with Bray-Curtis dissimilarity from MGS abundance data. All adjustments confirm a significant impact of the QASD score on microbiome composition within the adjusted dbRDA framework (FDR level).

**Supplementary Figure 6: Summary of Microbiome and Metabolome Alterations Across Pairwise Levels of the QASD Score**

Number of microbiome and metabolome features (y-axis) from different OMICs spaces (x-axis) showing significant variations across different pairwise levels of the QASD score, as determined by metadeconfoundR analyses. Features are color-coded based on the status of the association: **AD** (Ambiguously deconfounded), **C** (Confounded by corresponding variables), **OK_nc** (No other covariates), and **OK_sd** (Strictly deconfounded, the most stringent label, indicating that although another covariate is associated with the feature, the signal can still be attributed to the pairwise level of the lifestyle scores). PATHGROUPFINAL=Metacardis clinical groups variable.

**Supplementary Figure 7: MetaDeconfoundR Analysis of MGS and Serum Metabolites Across Individual Components of the QASD Score**

**(A)** Venn diagram showing overlaps among 141 metagenomic species (MGS) with strictly deconfounded status (OK_sd) based on intake of metformin, statin, PPI, and MetaCardis clinical groups according to metadeconfoundR results, comparing the extremes of the QASD score (high vs. low) and its individual components (FDR < 0.1, Cliff’s delta effect sizes > 0.1).

**(B)** Venn diagram showing overlaps among 19 MGS with strictly deconfounded status (OK_sd) under the same conditions as in (A), with Cliff’s delta effect sizes < -0.1. **(C)** Violin plots depicting the distribution of Cliff’s delta effect sizes for MGS in panels (A) and (B) across tested variables (x-axis). Numbers in brackets represent p-values from pairwise Wilcoxon rank-sum tests comparing the QASD score against its individual components. **(D-G)** Scatterplots of Cliff’s delta effect sizes for MGS in panels (A) and (B), comparing the QASD score (high vs. low; x-axis) with each of its individual components (y-axis). **(H)** Venn diagram showing overlaps among 371 serum metabolites with strictly deconfounded status (OK_sd) based on intake of metformin, statin, PPI, and MetaCardis clinical groups, comparing the extremes of the QASD score or its individual components (FDR < 0.1, Cliff’s delta effect sizes > 0.1). **(I)** Venn diagram showing overlaps among 315 serum metabolites with strictly deconfounded status (OK_sd) under the same conditions as in (H), with Cliff’s delta effect sizes < -0.1. **(J)** Violin plots showing the distribution of Cliff’s delta effect sizes for serum metabolites in panels (H) and (I) across tested variables (x-axis). Numbers in brackets correspond to p-values from pairwise Wilcoxon rank-sum tests (QASD score vs. its individual components). **(K-N)** Scatterplots of Cliff’s delta effect sizes for serum metabolites in panels (H) and (I), comparing the QASD score (high vs. low; x-axis) with its individual components (y-axis).

Positive Cliff’s delta values in Venn diagrams and scatterplots indicate MGS or serum metabolites enriched in individuals in the third tertile of the numerical variables included in the QASD score (e.g., AHEI, Diversity Simpson for all diet without beverages, total physical activity), in non-smokers, or in those with a high QASD score. Negative Cliff’s delta values indicate enrichment in individuals in the first tertile, in smokers, or in those with a low QASD score. Spearman's rho and the corresponding p-values are provided for each scatterplot, along with the fitted regression line. Points are colored according to the feature status in each metadeconfoundR analysis (OK_sd: Strictly deconfounded status, FDR < 0.01, absolute Cliff’s delta effect size > 0.1). Full results are available in Supplementary Table S8.

**Supplementary Figure 8: Summary of Significant Causal Mediation Relationships Between MGS and Serum Metabolites Across Extremes of the QASD Score**

Alluvial diagram illustrating the 3457 significant causal mediation relationships between the extremes of the QASD score (high vs. low; FDR < 0.05 for Average Causal Mediation Effect (ACME), Average Direct Effect (ADE), and Total effect) under same adjustment framework) as summarized in main Figure 6B. The diagram decomposes these relationships by the sign of the beta coefficients in three components: the QASD component of the ACME, the mediator component of the ACME, and the total effect of the QASD score on the dependent variable.

Positive mediations are defined as those where the sign of the mediator component of the ACME is positive (indicating a positive association between serum metabolites and MGS), while negative mediations have a negative mediator component (indicating a negative association between serum metabolites and MGS). Mediations are further stratified by the proportion of the mediation effect.

**Panels A and B** represent specific pairs of MGS and serum metabolites with significant mediation relationships in **Direction 1** (MGS as mediator, serum metabolite as the dependent variable) and **Direction 2** (serum metabolite as mediator, MGS as the dependent variable), respectively.
**Panels C and D** display shared pairs of MGS and serum metabolites with significant mediation relationships in both directions.

# Supplementary Results

**Supplemental results on links between diet and enterotypes**

Supplementary analyses confirmed and specified the links between diet and the prevalence of enterotypes. Adjusted logistic regression models by age, gender, BMI, center of recruitment, metformin, statin, and PPI intake showed significant associations between the prevalence of the different enterotypes with the QASD score and several nutritional factors (Supplemental Figure 4). The probability of Bact2 enterotype significantly decreases with higher levels of the QASD score (p-value=0.031 level 0-1-2 vs. level 5 and level 6-7, Supplemental Figure 4A) but also with the variety count of meat, fish and eggs (p-value=0.041, Supplemental Figure 4B), the intake of cookies and pastries (p-value=0.014, Supplemental Figure 4C), offal (p-value=9.68e-03, Supplemental Figure 4D) and vitamins like Biotin (p-value=0.027, Supplemental Figure 4E) and vitamin D (p-value=0.038, Supplemental Figure 4F). Conversely, the variety of dairy products (as assessed by Simpson score, p-value=0.041, Supplemental Figure 4G) and the intake of Vitamin A (p-value=0.048, Supplemental Figure 4H) were positively associated to a higher probability of the Prevotella enterotype. The intake of offals (p-value=0.044, Supplemental Figure 4D) and biotin (p-value=0.032, Supplemental Figure 4E) were also significantly associated to higher probability of the Prevotella enterotype in parallel with decrease probability of Bact2.

**Supplemental discussion literature on metagenomic and metabolomic biomarkers associated with QASD score**

At MGS level, high levels of the QASD score (5-6-7) were positively associated with multiple firmicutes lineages including 12 MGS belonging to *Faecalibacterium* genus and 2 MGS corresponding to *F. prausnitzii L2-6*, well known butyrate producing lineages positively associated to microbial gene richness and associated to healthy condition, depleted in different disease states ^1–3^ (Figure 4; Supplemental Table S8). *Eubacterium eligens*, and multiple MGS of the *Oscillibacter* and *Roseburia* genus were also enriched in individuals with high QASD score. These genera have been included in the healthy signature gut bacteria in the PredictUK study^4^, as well as *Ruminococcus lactaris*, a mucin-degrading commensal associated to healthy liver function together with *F. prausnitzii* ^5^. Also, high levels of the QASD score were associated with high levels of *Eubacterium ramulus*, a gut bacterium that has been proven to degrade flavonoids from buckwheat^6^. On the other hand, low levels of the QASD score (0-1-2-3) were associated to high levels of *Clostridium bolteae* and *Ruminococcus gnavus*, which are the microbes most strongly correlated with increased fasting and post-prandial inflammation and considered markers of poor health in the PredictUK study^4^ and markers of liver diseases^5^. At functional level, high QASD score levels (5-6-7) were associated with 6 Gut Metabolic Modules (GMM) corresponding to the degradation of aminoacids (phenylalanine, glutamate and lysine), lipids (triacylglycerol) and phenylpropanoid metabolism (phenylalanine and quinate degradation; Figure 4C).

At metabolomic level (Figure 5, Supplemental Table S9), high levels of the QASD score are positively associated with hippurate, multiple compounds derived from tryptophan metabolism (kynurenate, indoleacetate, indolepropionate, indoleacetylglutamine, indolin-2-one), acyl-cholines (palmitoylcholine, arachidonoylcholine, linoleoylcholine, dihomo−linolenoyl−choline, glycerophosphorylcholine, oleoylcholine, stearoylcholine), acyl-carnitines (triglylcarnitine, ximeloylcarnitine, methylsuccinoylcarnitine), chemical compounds associated to coffee consumption/metabolism (caffeine, 1−methylxanthine, 1,3,7−trimethylurate, 5−acetylamino−6−formylamino−3−methyluracil, 1,3-dimethylurate, homostachydrine) or pyrimidine related metabolites (uracil, uridine). Urine levels of Hippurate have been shown positively associated with microbial gene richness, improving glucose tolerance and enhancing insulin secretion in mouse experiments^7^. Indolepropionate (IPA) is a well-known microbial metabolite produced from dietary tryptophan that is predictive of the onset of T2D, with high levels associated to lower likelihood of developing T2D^8^ and regulates gastrointestinal barrier function notably via its interaction with the pregnane X receptor (PXR)^9^, being strongly associated with intake of dietary fiber and positively associated with microbiome diversity in the TwinsUK cohort^10,11^. Indoleacetate also belongs to tryptophan-derived microbial metabolites known to activate the nuclear receptor AhR, being depleted in metabolic syndrome^12^ and reduced in mice on a HFD versus a low-fat diet, attenuating cytokine-mediated lipogenesis in hepatocytes^13^, whereas kynurenate, an intermediate of kynurenine pathway of tryptophan degradation, plays an important role in cognition by acting on NMDA receptors^14,15^. Acyl cholines (choline acylated with a fatty acid) includes the well-known neurotransmitter acetylcholine but also long-chain unsaturated acylcholines detected in tissues of blood vessels obtained from the operations of patients suffering from cardiovascular diseases. Arachidonoylcholine and other unsaturated fatty acid analogs with the chain length of 18 and 22 carbon atoms are inhibitors of the neuronal and muscle-type nicotinic receptors and modest inhibitors of the acetylcholinesterase (AChE, EC 3.1.1.7) and butyrylcholinesterase (BChE, EC 3.1.1.8), and thus could act as endogenous modulators of the acetylcholine signaling system^16^. Palmitoylcholine has been also associated to T2D^17^. In relation with pyrimidine-related metabolites, uracil-induced immune response is required for efficient elimination of bacteria, intestinal cell repair, and host survival during infection of nonresident species^18^, whereas uridine supplementation reduced the body weight and suppressed the accumulation of subcutaneous, epididymal, and mesenteric white adipose tissue in high fat diet-fed mice, attenuating HFD-induced obesity and nonalcoholic fatty liver disease^19^. Finally, coffee consumption has been associated with reduction of the risk of chronic diseases such as metabolic syndrome, obesity, type 2 diabetes, cardiovascular diseases, or some types of cancer^20–22^, and has been reported positively associated to microbiome diversity in individuals of the US Arrivale cohort^23^, in line with our results in terms of positive association with microbial gene richness (Figure 1A).

On the opposite side, low levels of the QASD score (0-1-2-3) are positively associated to different branched-chain aminoacids and related metabolites (2−hydroxy−3−methylvalerate, isobutyrate, (r)-3- oxybutyrylcarnitine,alpha-hydroxyisovalerate,N-acetylisoleucine), bile-acid related metabolites (taurocholenate sulfate, tauroursodeoxycholate, glycoursodeoxycholate, glycochenodeoxycholate glucuronide, isoursodeoxycholate sulphate, glycochenodeoxycholate 3−sulfate, taurochenodeoxycholate), markers of tobacco consumption (norcotinine, cotinine, hydroxycotinine) and different dipeptides (gamma glutamylleucine, gamma−glutamylvaline, gamma-glutamylisoleucine). Increased BCAA levels has been observed in diabetes type 2 and obesity^24–26^, whereas Gamma-Glutamyl peptides have been identified as potential biomarkers for a number of diseases including cancer, diabetes, and liver disease. For example, gamma glutamyl-leucine has been extensively reported to be associated with the risk of cardio-metabolic diseases, such as obesity, metabolic syndrome, and type 2 diabetes^27,28^, whereas gamma-glutamyl-isoleucine has been reported as potential biomarker for prostate cancer^29^. However, gamma−glutamyl-valine, which is also enriched in individuals with low QASD score levels, is a naturally occurring bioactive dipeptide primarily found in foods such as legumes (i.e., common beans, soybeans, black gram), garlic, onions, cheese, and other fermented foods^30^ that has been characterized as an anti-inflammatory peptide, with in-vitro anti-inflammatory activities against vascular, gastrointestinal, and adipocyte inflammation^31–34^. Isoursodeoxycholate sulfate has been recently identified a serum biomarker associated to post-prandial lipemia, inflammation and worse hepatic function and anticorrelated with Shannon diversity in human gut microbiome^35,36^, whereas increased levels of glycochenodeoxycholate 3−sulfate has been observed in patients with Crohn disease and in bariatric surgery patients suffering from gallstones^37,38^. Finally, among the compounds enriched in individuals with low QASD score we found also GlycA, a novel spectroscopic marker of systemic inflammation with low intra-individual variability and other attributes favoring its clinical use in patients with chronic inflammatory and autoimmune diseases^39^, 2-hydroxybutyrate, a biomarker of T2D derived from protein metabolism whose levels increases in parallel with the progression of insulin resistance^40^, but also azelate, a compound occurring naturally in organisms ranging from plants to humans with a broad range of immunomodulatory activities in vitro and in vivo that can mitigate insulin resistance^41^.

**References**

1. Lopez-Siles, M., Duncan, S. H., Garcia-Gil, L. J. & Martinez-Medina, M. Faecalibacterium prausnitzii: from microbiology to diagnostics and prognostics. *ISME J.* **11**, 841–852 (2017).

2. Munukka, E. *et al.* Faecalibacterium prausnitzii treatment improves hepatic health and reduces adipose tissue inflammation in high-fat fed mice. *ISME J.* **11**, 1667–1679 (2017).

3. Sokol, H. *et al.* Faecalibacterium prausnitzii is an anti-inflammatory commensal bacterium identified by gut microbiota analysis of Crohn disease patients. *Proc. Natl. Acad. Sci. U. S. A.* **105**, 16731–16736 (2008).

4. Asnicar, F. *et al.* Microbiome connections with host metabolism and habitual diet from 1,098 deeply phenotyped individuals. *Nat. Med.* (2021) doi:10.1038/s41591-020-01183-8.

5. Li, R.-J. *et al.* Network of Interactions Between Gut Microbiome, Host Biomarkers, and Urine Metabolome in Carotid Atherosclerosis. *Front. Cell. Infect. Microbiol.* **11**, 708088 (2021).

6. Simmering, R., Pforte, H., Jacobasch, G. & Blaut, M. The growth of the flavonoid-degrading intestinal bacterium, Eubacterium ramulus, is stimulated by dietary flavonoids in vivo. *FEMS Microbiol. Ecol.* **40**, 243–248 (2002).

7. Brial, F. *et al.* Human and preclinical studies of the host–gut microbiome co-metabolite hippurate as a marker and mediator of metabolic health. *Gut* **70**, 2105–2114 (2021).

8. de Mello, V. D. *et al.* Indolepropionic acid and novel lipid metabolites are associated with a lower risk of type 2 diabetes in the Finnish Diabetes Prevention Study. *Sci. Rep.* **7**, 46337 (2017).

9. Venkatesh, M. *et al.* Symbiotic Bacterial Metabolites Regulate Gastrointestinal Barrier Function via the Xenobiotic Sensor PXR and Toll-like Receptor 4. *Immunity* **41**, 296–310 (2014).

10. Pallister, T. *et al.* Characterizing Blood Metabolomics Profiles Associated with Self-Reported Food Intakes in Female Twins. *PLOS ONE* **11**, e0158568 (2016).

11. Menni, C. *et al.* Circulating levels of the anti-oxidant indoleproprionic acid are associated with higher gut microbiome diversity. *Gut Microbes* **10**, 688–695 (2019).

12. Natividad, J. M. *et al.* Impaired Aryl Hydrocarbon Receptor Ligand Production by the Gut Microbiota Is a Key Factor in Metabolic Syndrome. *Cell Metab.* **28**, 737-749.e4 (2018).

13. Krishnan, S. *et al.* Gut Microbiota-Derived Tryptophan Metabolites Modulate Inflammatory Response in Hepatocytes and Macrophages. *Cell Rep.* **23**, 1099–1111 (2018).

14. Kennedy, P. J., Cryan, J. F., Dinan, T. G. & Clarke, G. Kynurenine pathway metabolism and the microbiota-gut-brain axis. *Neuropharmacology* **112**, 399–412 (2017).

15. Stone, T. W. & Darlington, L. G. The kynurenine pathway as a therapeutic target in cognitive and neurodegenerative disorders: Kynurenines and CNS disorders. *Br. J. Pharmacol.* **169**, 1211–1227 (2013).

16. Akimov, M. G. *et al.* Arachidonoylcholine and Other Unsaturated Long-Chain Acylcholines Are Endogenous Modulators of the Acetylcholine Signaling System. *Biomolecules* **10**, 283 (2020).

17. Yousri, N. A. *et al.* Metabolic and Metabo-Clinical Signatures of Type 2 Diabetes, Obesity, Retinopathy, and Dyslipidemia. *Diabetes* **71**, 184–205 (2022).

18. Lee, K.-A. *et al.* Bacterial-Derived Uracil as a Modulator of Mucosal Immunity and Gut-Microbe Homeostasis in Drosophila. *Cell* **153**, 797–811 (2013).

19. Liu, Y. *et al.* Uridine attenuates obesity, ameliorates hepatic lipid accumulation and modifies the gut microbiota composition in mice fed with a high-fat diet. *Food Funct.* **12**, 1829–1840 (2021).

20. Farias-Pereira, R., Park, C.-S. & Park, Y. Mechanisms of action of coffee bioactive components on lipid metabolism. *Food Sci. Biotechnol.* **28**, 1287–1296 (2019).

21. Grosso, G., Godos, J., Galvano, F. & Giovannucci, E. L. Coffee, Caffeine, and Health Outcomes: An Umbrella Review. *Annu. Rev. Nutr.* **37**, 131–156 (2017).

22. O’Keefe, J. H., DiNicolantonio, J. J. & Lavie, C. J. Coffee for Cardioprotection and Longevity. *Prog. Cardiovasc. Dis.* **61**, 38–42 (2018).

23. Manor, O. *et al.* Health and disease markers correlate with gut microbiome composition across thousands of people. *Nat. Commun.* **11**, 5206 (2020).

24. Carlsten, A., Hallgren, B., Jagenburg, R., Svanborg, A. & Werkö, L. Amino Acids and Free Fatty Acids in Plasma in Diabetes: I. The Effect of Insulin on the Arterial Levels. *Acta Med. Scand.* **179**, 361–370 (2009).

25. She, P. *et al.* Obesity-related elevations in plasma leucine are associated with alterations in enzymes involved in branched-chain amino acid metabolism. *Am. J. Physiol.-Endocrinol. Metab.* **293**, E1552–E1563 (2007).

26. Kuzuya, T. *et al.* Regulation of branched-chain amino acid catabolism in rat models for spontaneous type 2 diabetes mellitus. *Biochem. Biophys. Res. Commun.* **373**, 94–98 (2008).

27. Wu, Q. *et al.* Gamma-glutamyl-leucine levels are causally associated with elevated cardio-metabolic risks. *Front. Nutr.* **9**, 936220 (2022).

28. Li, J. *et al.* Tyrosine and Glutamine-Leucine Are Metabolic Markers of Early-Stage Colorectal Cancers. *Gastroenterology* **157**, 257-259.e5 (2019).

29. Thacker, J. B., He, C. & Pennathur, S. Quantitative analysis of γ‐glutamylisoleucine, γ‐glutamylthreonine, and γ‐glutamylvaline in HeLa cells using UHPLC‐MS/MS. *J. Sep. Sci.* **44**, 2898–2907 (2021).

30. Yang, J., Bai, W., Zeng, X. & Cui, C. Gamma glutamyl peptides: The food source, enzymatic synthesis, kokumi-active and the potential functional properties – A review. *Trends Food Sci. Technol.* **91**, 339–346 (2019).

31. Guha, S., Alvarez, S. & Majumder, K. Transport of Dietary Anti-Inflammatory Peptide, γ-Glutamyl Valine (γ-EV), across the Intestinal Caco-2 Monolayer. *Nutrients* **13**, 1448 (2021).

32. Zhang, H., Kovacs-Nolan, J., Kodera, T., Eto, Y. & Mine, Y. γ-Glutamyl cysteine and γ-glutamyl valine inhibit TNF-α signaling in intestinal epithelial cells and reduce inflammation in a mouse model of colitis via allosteric activation of the calcium-sensing receptor. *Biochim. Biophys. Acta BBA - Mol. Basis Dis.* **1852**, 792–804 (2015).

33. Xing, L., Zhang, H., Majumder, K., Zhang, W. & Mine, Y. γ-Glutamylvaline Prevents Low-Grade Chronic Inflammation via Activation of a Calcium-Sensing Receptor Pathway in 3T3-L1Mouse Adipocytes. *J. Agric. Food Chem.* **67**, 8361–8369 (2019).

34. Guha, S., Paul, C., Alvarez, S., Mine, Y. & Majumder, K. Dietary γ-Glutamyl Valine Ameliorates TNF-α-Induced Vascular Inflammation *via* Endothelial Calcium-Sensing Receptors. *J. Agric. Food Chem.* **68**, 9139–9149 (2020).

35. Wilmanski, T. *et al.* *Blood Metabolome Signature Predicts Gut Microbiome α-Diversity in Health and Disease*. http://biorxiv.org/lookup/doi/10.1101/561209 (2019) doi:10.1101/561209.

36. Louca, P. *et al.* The secondary bile acid isoursodeoxycholate correlates with post-prandial lipemia, inflammation, and appetite and changes post-bariatric surgery. *Cell Rep. Med.* **4**, 100993 (2023).

37. Feng, L. *et al.* Co‐occurrence of gut microbiota dysbiosis and bile acid metabolism alteration is associated with psychological disorders in Crohn’s disease. *FASEB J.* **36**, (2022).

38. Guman, M. S. S. *et al.* Adipose Tissue, Bile Acids, and Gut Microbiome Species Associated With Gallstones After Bariatric Surgery. *J. Lipid Res.* **63**, 100280 (2022).

39. Connelly, M. A., Otvos, J. D., Shalaurova, I., Playford, M. P. & Mehta, N. N. GlycA, a novel biomarker of systemic inflammation and cardiovascular disease risk. *J. Transl. Med.* **15**, 219 (2017).

40. Sousa, A. P. *et al.* Which Role Plays 2-Hydroxybutyric Acid on Insulin Resistance? *Metabolites* **11**, 835 (2021).

41. Izbicka, E. & Streeper, R. T. Azelaic Acid Esters as Pluripotent Immunomodulatory Molecules: Nutritional Supplements or Drugs. *Nutraceuticals* **1**, 42–53 (2021).
